# Aboveground plant-to-plant electrical signaling mediates network acquired acclimation

**DOI:** 10.1093/plcell/koac150

**Published:** 2022-05-20

**Authors:** Magdalena Szechyńska-Hebda, Maria Lewandowska, Damian Witoń, Yosef Fichman, Ron Mittler, Stanisław M Karpiński

**Affiliations:** Department of Plant Genetics, Breeding and Biotechnology, Institute of Biology, Warsaw University of Life Sciences, 02-776 Warsaw, Poland; Department of Plant Genetics, Breeding and Biotechnology, Institute of Biology, Warsaw University of Life Sciences, 02-776 Warsaw, Poland; Department of Plant Genetics, Breeding and Biotechnology, Institute of Biology, Warsaw University of Life Sciences, 02-776 Warsaw, Poland; The Division of Plant Sciences and Technology and Interdisciplinary Plant Group, College of Agriculture, Food and Natural Resources, Christopher S. Bond Life Sciences Center, University of Missouri, Columbia, Missouri 65201, USA; The Division of Plant Sciences and Technology and Interdisciplinary Plant Group, College of Agriculture, Food and Natural Resources, Christopher S. Bond Life Sciences Center, University of Missouri, Columbia, Missouri 65201, USA; Department of Plant Genetics, Breeding and Biotechnology, Institute of Biology, Warsaw University of Life Sciences, 02-776 Warsaw, Poland

## Abstract

Systemic acquired acclimation and wound signaling require the transmission of electrical, calcium, and reactive oxygen species (ROS) signals between local and systemic tissues of the same plant. However, whether such signals can be transmitted between two different plants is largely unknown. Here, we reveal a new type of plant-to-plant aboveground direct communication involving electrical signaling detected at the surface of leaves, ROS, and photosystem networks. A foliar electrical signal induced by wounding or high light stress applied to a single dandelion leaf can be transmitted to a neighboring plant that is in direct contact with the stimulated plant, resulting in systemic photosynthetic, oxidative, molecular, and physiological changes in both plants. Furthermore, similar aboveground changes can be induced in a network of plants serially connected via touch. Such signals can also induce responses even if the neighboring plant is from a different plant species. Our study demonstrates that electrical signals can function as a communication link between transmitter and receiver plants that are organized as a network (community) of plants. This process can be described as network-acquired acclimation.

## Teaser

A new type of plant-to-plant direct communication involving electrical signaling, reactive oxygen species, and photosystem networks is revealed.

In a Nutshell **Background:** An injured leaf (e.g. by insect herbivory or excess light) generates electric signals (ES) that spread to tissues, leaves, and organs of the entire plant. ES are mediated by changes in the activity of ion channels and are accompanied by waves of reactive oxygen species (ROS) and nonphotochemical quenching (NPQ). These waves are interdependent and propagate systemically throughout the plant. This process is essential for priming specific changes in gene expression and plant acclimation (e.g. cellular light memory). As a result, the entire plant enters a state of systemic acquired acclimation (SAA).

**Question:** Could ES, ROS, and NPQ waves spread from an injured plant to neighboring plants, if their leaves touch? Until now, only indirect signaling routes like underground ES mediated by mycorrhizal networks between roots of different plants, or aboveground plant volatiles, were found to connect different plants.

**Findings:** Under humid conditions, an injured plant can directly communicate a danger signal to other plants that touch it within a community of plants, like a meadow of dandelions. ES and ROS waves serve as plant-to-plant signals propagating on and in the leaf with a velocity of several millimeters per second or centimeters per minute, respectively. These signals can induce changes in NPQ, chloroplast retrograde signaling, gene expression, phytohormones, ROS signaling, and acclimation responses, in neighboring plants. Most of these complex communication responses can also be induced between two plants connected by a copper wire circuit, indicating that ES is the main player of plant-to-plant communication. We show that ES and ROS induce a new acclimation phenomenon termed “network acquired acclimation (NAA),” necessary for SAA induction within a plant community.

**Next steps:** NAA may be considered as a new communication mechanism within plant ecosystems. However, whether NAA is a “side effect” of SAA, or confers an evolutionary advantage to plants living within the community, and to what extent can ES carry specific information and determine specific responses, are open questions.

## Introduction

Signaling is the transfer of information in the form of an action generating changes in the *status quo* of a system in a direction from a transmitter unit to a receiver unit. Communication occurs when the information transmitted induces a response in the receiver. Land plants can communicate at the level of cells, tissues, and organs, using the flow of electric signals (ESs) and hydraulic waves (physical signals), as well as phytohormones, reactive oxygen species (ROS), calcium (Ca^2+^), peptides, and micro RNAs (chemical signals). ES can transmit information more quickly over long distances from the stressed zone to other parts of the plant body when compared with chemical signals (Szechyńska-Hebda et al., 2010; Gilroy et al., 2016; Białasek et al., 2017; Kurenda et al., 2019, Farmer et al., 2020; G�recka et al., 2020; Vega-Mu�oz et al., 2020; Fichman and Mittler, 2021; Sukhova and Sukhov, 2021). Since ES were first discovered in the Venus flytrap (*Dionaea muscipula*) (Burdon-Sanderson, 1873), various types of ES have been described, triggered by different stimuli and spreading within a single plant (Zimmermann et al., 2009; Karpiński and Szechyńska-Hebda, 2010; Salvador-Recatal� et al., 2014; Nguyen et al., 2018; Kurenda et al., 2019). The information encoded in the spatial and temporal dynamics of ES evokes plant-wide responses such as changes in photosynthesis, nonphotochemical and photochemical quenching (NPQ and qP), gas exchange, phloem transport, gene expression, protein synthesis, and systemic acquired acclimation (SAA) (Karpinski et al., 1999; Pogson et al., 2008; Szechyńska-Hebda et al., 2010; Furch et al., 2010; Gilroy et al., 2016; Sukhov, 2016; Białasek et al., 2017; Toyota et al., 2018; G�recka et al., 2020; Sukhova and Sukhov, 2021).

Indirect (not requiring physical contact) signaling between plants has been shown to occur underground between roots, mediated by the mycorrhizal network (Song et al., 2019). Electrostimulation can serve as a trigger for root communication within the plant-wide web regardless if plants are the same or different species (Volkov et al., 2019). Indirect signaling through aboveground emission of plant volatile molecules (Riedlmeier et al., 2017) can also change gene expression and the synthesis of defense metabolites in neighboring plants. However, a more direct aboveground exchange of information between plants could provide a highly important mechanism synchronizing defense and acclimation responses within a plant network during stressful events. We, therefore, addressed the question of whether plants that live as a community, like dandelion (*Taraxacum officinale*) or Arabidopsis (*Arabidopsis thaliana*), can use canopy-wide ES to communicate with each other. Our results strongly suggest that direct aboveground ES transmission between a stressed transmitter plant and an unstressed receiver plant is driving spatial changes in the distribution of photosynthetic energy and induction of common signaling and defense molecules in the receiver plant, and that this type of plant-to-plant aboveground communication can occur in a community of plants to induce network acquired acclimation (NAA).

## Results

### Injury-induced physiological responses in a dandelion leaf

NPQ, a parameter of chlorophyll (Chl) *a* fluorescence, describes a phenomenon mediated by the trans-thylakoidal pH gradient and the proton sensor protein PsbS, whereby excess energy absorbed by the photosynthetic apparatus is dissipated in the form of heat (quenched) to prevent ROS generation and photosynthetic inactivation (Baker, 2008; Szechyńska-Hebda et al., 2017; G�recka et al., 2020). We observed that an increase in NPQ initiated at the site of heat injury can spread to distal parts of a dandelion leaf (Figure�1 and Supplemental Table S1), first via vein tissues, and then into the mesophyll cells between veins (Figure�1, A and B). Comparing NPQ levels across different leaf areas revealed the wave-like nature of this NPQ change (Figure�1, C and D), which corresponds to two components of photosynthetic inactivation: fast and long-term inactivation (Figure�1D, first and second NPQ maxima, respectively). However, the amplitude of changes decreased, while their periods increased, with increasing distance from the heated spot (Figure�1, E and F).

**Figure 1:**
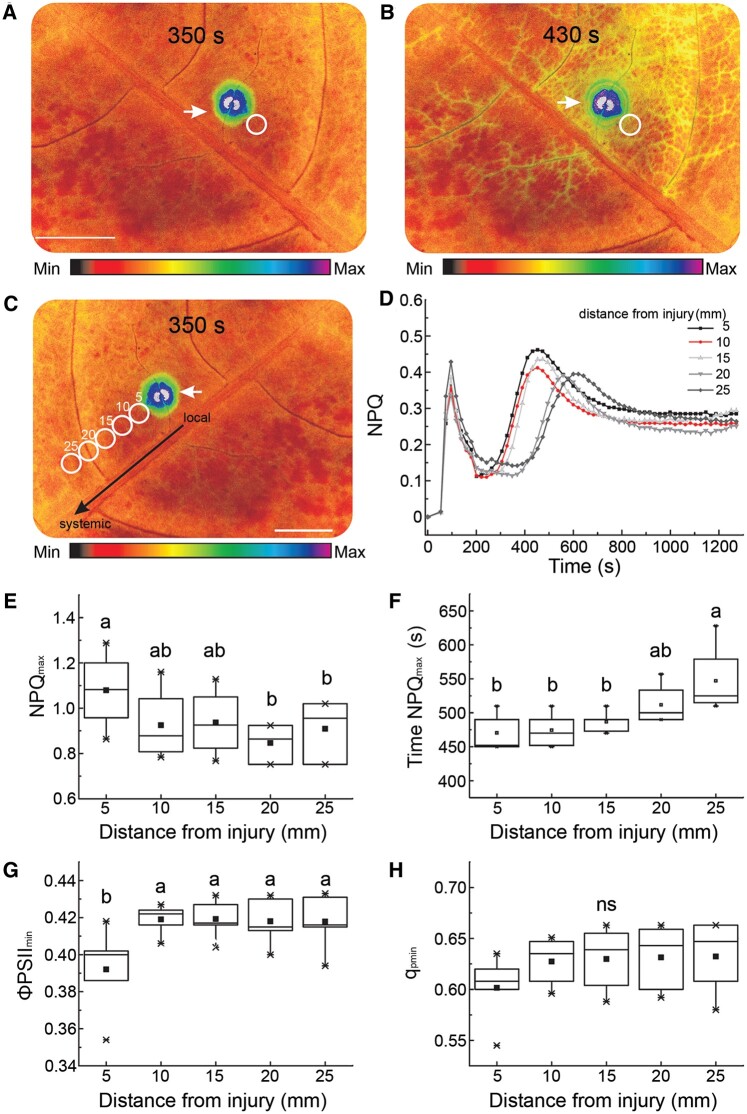
Changes in photosynthetic parameters in dandelion leaves following a heat injury. A–C, Representative images of the spatiotemporal changes in NPQ after heat injury, at 350 s, and 430 s into the recording (full quantified results in [D]). White circles correspond to distances from the site of injury: 5, 10, 15, 20, and 25 mm. Color scales represent the range of values of the measured parameter. The injured zone is indicated by an arrow. Scale bars = 1 cm. D, NPQ kinetics as a function of distance from the heat-injured zone (normalized records for circled areas in [C]). The two NPQ maxima correspond to two components of photosynthetic inactivation*:* fast inactivation and long-term inactivation (*n* = 5). E, Quantification of maximum NPQ peak (amplitude). F, Period of maximum NPQ peak. G, Quantum use efficiency of PSII ΦPSII. H, qP of PSII. E–H, Parameters were measured in each analyzed area (circles in [C]), and correspond to long-term responses. Different lowercase letters indicate significant differences (*P* < 0.05) as determined by ANOVA analysis followed by a Tukey’s test; ns, not significant. Data are shown as means � se (*n* = 10).

In contrast, quantum use efficiency of photosystem II (ΦPSII), and qP of PSII, increased exponentially with increasing distance from the wounded site (Figure�1, G and H, long-term responses). These responses were similar to published results in other plants following stress application (Szechyńska-Hebda et al., 2010; Białasek et al., 2017; G�recka et al., 2020). We also determined that the short temperature rise induced by the point injury does not substantially contribute to systemic NPQ changes (Supplemental Figure S1, A–D). The mechanism of NPQ change thus appeared to be paramount for the observed effect, as leaf temperature decreased below the initial value (Supplemental Figure S1C), even though other foliar cooling mechanisms such as transpiration dropped by 25%–40%, for at least 120 min following heat injury relative to noninjured plants (Supplemental Figure S1, E–G and Supplemental Movie S1).

### Plant-to-plant aboveground signal transduction and communication

To test whether NPQ and other responses to a local heat stimulus can be transmitted between plants, we connected two different plants (one transmitter and one receiver) by their leaves (touching each other). To induce high relative humidity and leaf-to-leaf conductivity, we sprayed plants with water (Figure�2;Supplemental Figure S2 and Supplemental Movie S2), or connected their leaves with a drop of agarose (Supplemental Figure S3). Indeed, localized heat injury of the transmitter plant triggered a systemic increase in NPQ (Figure�2, A–C) and foliar ROS (Figure�2, D and E and Supplemental Figure S2) levels within both the transmitter and receiver plants. There were no significant changes in control plants connected under the same conditions, but not injured (Figure�2A). We observed that the kinetics of NPQ and ROS changes for the transmitter and receiver plants (Figure�2, C and E) are similar. Systemic ROS accumulation reached higher levels in the receiver compared to the transmitter plant, with the signal being transmitted even though we applied the injury to a leaf other than the one touching the receiver plant (Figure�2, D and E and Supplemental Figure S2). Correspondingly, NPQ and the slope coefficient of the linear function fitted to the NPQ curve (Supplemental Figure S3, A–E) had a higher value for the receiver plant than the control plant in the system using agarose-connected plants.

**Figure 2:**
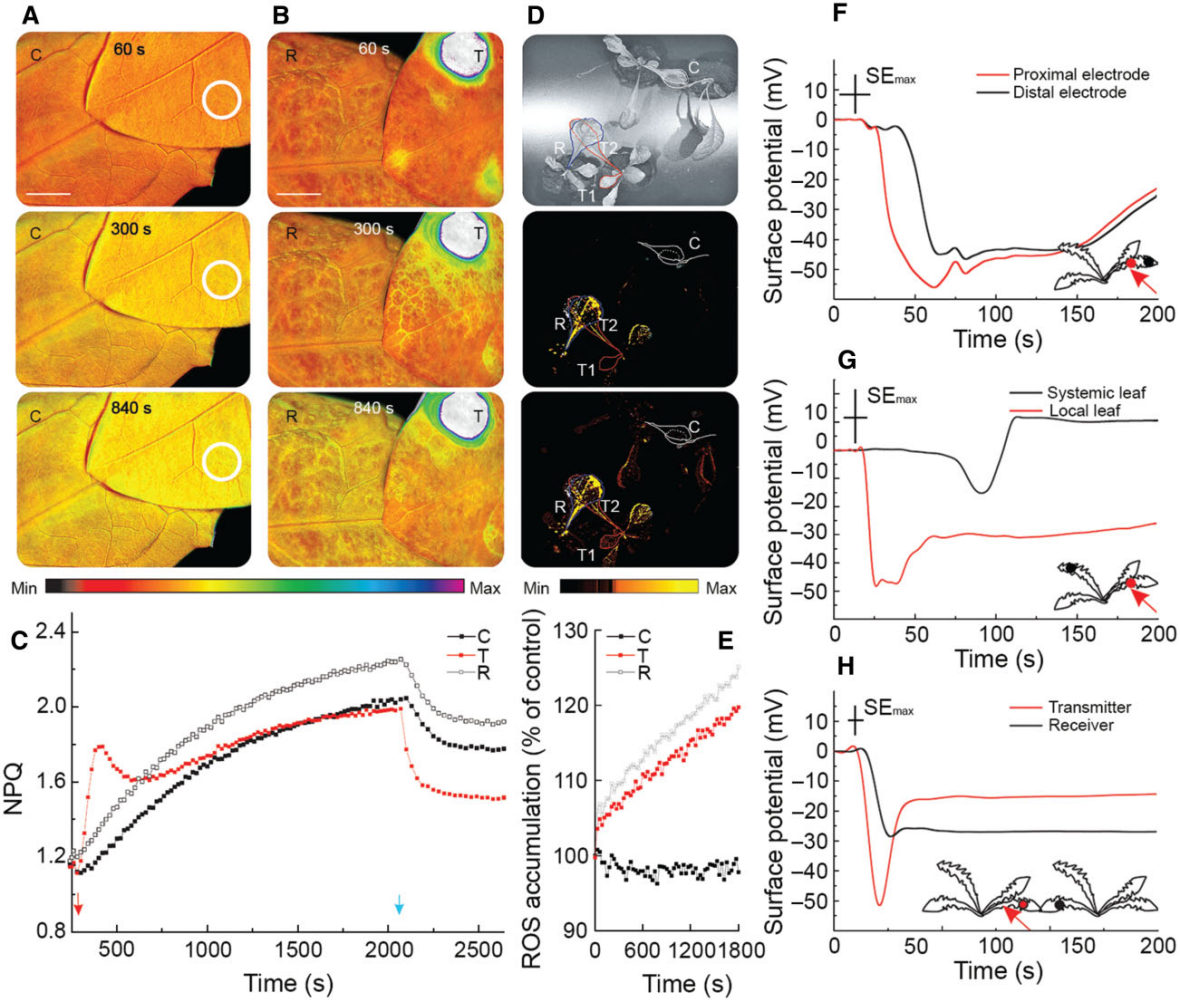
Plant-to-plant transmission of NPQ, ROS, and ES detected at the surface of dandelion leaves following a heat injury. Leaves of two different dandelion plants were connected by a simple touch following spraying with water to ensure conductivity. A, Time lapse imaging of NPQ for a representative pair of control (C) leaves, at 60, 300, and 840 s after one leaf was touched with an unheated metal wire (circles). B, Time lapse imaging of NPQ for a pair of transmitter (T) and receiver (R) leaves, at 60, 300, and 840 s after the T leaf was touched with a heated metal wire for 2 s (spot). Scale bar = 1 cm. Color scales represent the range of values of the measured parameter. C, Quantification of NPQ in C, T, and R plants. In (A–C), plants were dark-adapted for at least 20 min before imaging. Heat injury was applied at 330 s (arrow). NPQ was measured under actinic light over 1,630 s, and then the light was switched off (arrow) for the next 300 s. D, Whole-plant ROS imaging of two pairs of touching plants with H_2_DCFDA. T1, locally injured transmitter leaf; T2, transmitter systemic leaf; R, receiver leaf; C, control uninjured pair of plants. Representative imaging at 0, 1,200, and 1,800 s are shown. Color scales represent the range of values of the measured parameter. (E) Quantification of ROS fluorescence in C, R, and T2 plants shown in (D). F, ESs recorded for one leaf by two electrodes separated by ∼10 mm. G, ES recorded for different leaves of the same plant (a local injured and a systemic untreated leaf); the measuring electrodes were separated by ∼140 mm. H, ES recorded for leaves of two different plants (injured T and untreated R) touching each other; the measuring electrodes were separated by ∼30 mm. F–H, Relative ES values were measured in I = 0 mode, and representative recordings are presented (normalized records). Arrows indicate the place of injury, applied at 10 s. Red dots, electrodes located near the injury place; black dots, electrodes located distantly on the same leaf, or on another leaf of the same plant, or on leaf of different plant. Maximal SE is indicated for the x-axis (time) and y-axis (surface potential) by the length of the crossed error bars.

The dark relaxation of the proton gradient and proton conductivity across the thylakoid membrane may be accelerated with signal transduction (Sukhov et al., 2016). An exponential function of the dark relaxation of NPQ showed the fastest changes with the transmitter plants (25 s), compared to the slower changes seen for the receiver plant (46 s), with the slowest change observed in unheated control plants (62 s) (Supplemental Figure S3, C and F and Supplemental Table S2). Compared to earlier results collected on pea (*Phaseolus vulgaris*) leaves (Sukhov et al., 2016), the above results may illustrate a new mechanism by which ES influences photosynthesis that is not based on proton influx or changes in extracellular and intracellular pH. The ΦPSII and qP kinetics were inversely related to the changes in NPQ (Supplemental Figure S4). Further noninvasive extracellular measurements of relative electrical potential supported the existence of an injury-induced ES on the leaf surface (Figure�2, F–H and Supplemental Figure S5). Touching a transmitter leaf with a heated metal stick generated an ES, which began as a small (several microvolts) and transient membrane hyperpolarization. The ES then exhibited a rapid (within a few seconds) depolarization phase with an amplitude in the range of –25 to –55 mV in the transmitter plant, and in the range of –5 to –25 mV in the receiver (Figure�2, F–H and Supplemental Figure S5, A–C). Some pulses of depolarization with a period of ∼50 s also occurred within the slow repolarization phase (Figure�2, F–H). The ES moved within different areas of the same leaf (Figure�2F), from organ to organ (Figure�2G), and importantly, from plant to plant (Figure�2H). However, the ES weakened with increasing distance from the site of injury (Figure�2, F–H). Although the ES amplitude decreased with increasing distance from the injury site, it was independent of the direction of signal transduction, that is within or between plants, as recorded with electrode pairs at various distances ranging from 10 mm to 140 mm (Figure�2, F–H and Supplemental Figure S5). Using the Gaussian model, we calculated an ES propagation velocity of 5 mm s^−1^ between different plants (Supplemental Figure S5B). The ES signal from the injured transmitter plant was transferred to a receiver plant, but only when a wet contact between leaves was established, whereas ES did not propagate between dry touching leaves (Supplemental Figure S5A). Importantly, the transition of all signal types (NPQ, ROS, and ES) was not restricted to parastichous leaves (with developmentally determined direct vascular connections between leaves; according to the formula *n* + 5 and *n* + 8).

### Inhibition of plant-to-plant signal transduction

ES has previously been reported to be involved in the systemic regulation of ROS, NPQ, and photosynthesis, as has the inhibition of ES and ROS signal by lanthanum chloride (LaCl_3_), a nonspecific Ca^2+^ antagonist that can be used as an inhibitor of Ca^2+^-dependent signaling, within a plant (Miller et al., 2009; Białasek et al., 2017; Tian et al., 2020). To test whether ES and communication between two directly touching plants (transmitter and receiver) depended on ion changes at the level of the leaf surface, with particular emphasis on the role of Ca^2+^ ions, we connected the transmitter leaf to the receiver plant with a drop of a 2-mM LaCl_3_ solution prior to wounding (Figure�3). Under these conditions, we observed the inhibition of ROS accumulation in the two touching dandelion plants after wounding a single leaf on one plant, compared with plant leaves connected with a drop of water only.

**Figure 3:**
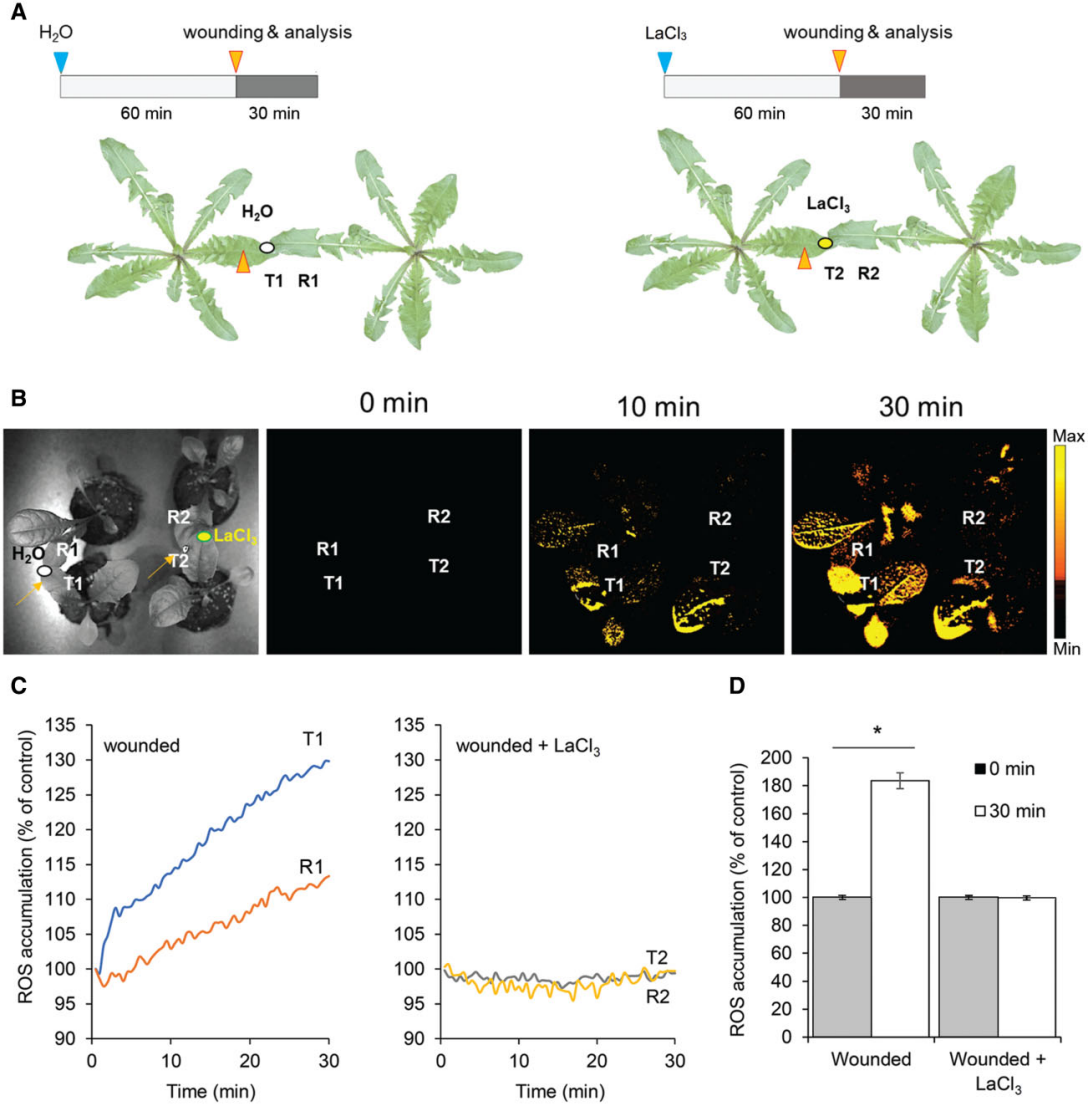
Inhibition of plant-to-plant ROS signaling by a LaCl_3_. Schematic diagrams of the experiments presented in (B)–(D). Leaves of two different dandelion plants were connected with a drop of water (ellipse) or a drop of a 2-mM LaCl_3_ solution (a Ca^2+^ antagonist that can be used as an inhibitor of Ca^2+^-dependent signaling; ellipse) to ensure conductivity 60 min prior to wounding. In the pair of connected leaves, one leaf (transmitter, indicated as T1, T2) was touched with a heated metal wire, while the second leaf (receiver, indicated as R1, R2) was untreated. Wounding is indicated by arrows on the leaf surface. B, Whole-plant ROS imaging of two pairs of touching plants, as determined by H_2_DCFDA fluorescence; time lapse images are representative of 0, 10, and 30 min after single-leaf injury. Color scales represent the range of values of the measured parameter. C, Quantification of ROS fluorescence in water- and LaCl_3_-connected plants. D, Comparison of ROS fluorescence in R leaves before and 30 min postwounding. *P < 0.05 indicates a significant difference between the time 0 min and time 30 min, as determined by Student’s *t* test. Data are shown as means � se (*n* = 8).

### Electric currents play a key role in aboveground plant-to-plant communication

To determine the relative roles of electric and ROS signals in aboveground plant-to-plant communication, we connected two leaves from different plants with a copper wire in an enclosed direct current (DC) circuit. When the transmitter plant was injured with a heated metal stick, we observed similar spatiotemporal changes for NPQ in both injured transmitter and untreated receiver plants (Supplemental Figure S6). To exclude a possible contribution from volatile plant signaling (Song et al., 2019), we exposed a transmitter plant kept within an enclosed transparent box to a bright blue light laser (2,000 �mol photons –^2^ s^−1^, 445 nm) (Figure�4). ES originating at the laser-injured leaf transduced from the transmitter plant via the copper wire to the receiver plant that was enclosed in a different plastic box with black walls, thus protected from the side effect of the laser. The ES generated by the transmitter plant induced significant (*P* ≤ 0.01) changes in the ratio between variable and minimal fluorescence (*F*_v_/*F*_o_), the operational quantum efficiency of PSII in the light-adapted state (*F*_v_′/*F*_m_′), and the Chl fluorescence decrease ratio (Rfd). We did not observe such changes in control experiments in which plants in the different boxes were connected with a wire but were not injured with the laser (Supplemental Figure S7).

**Figure 4:**
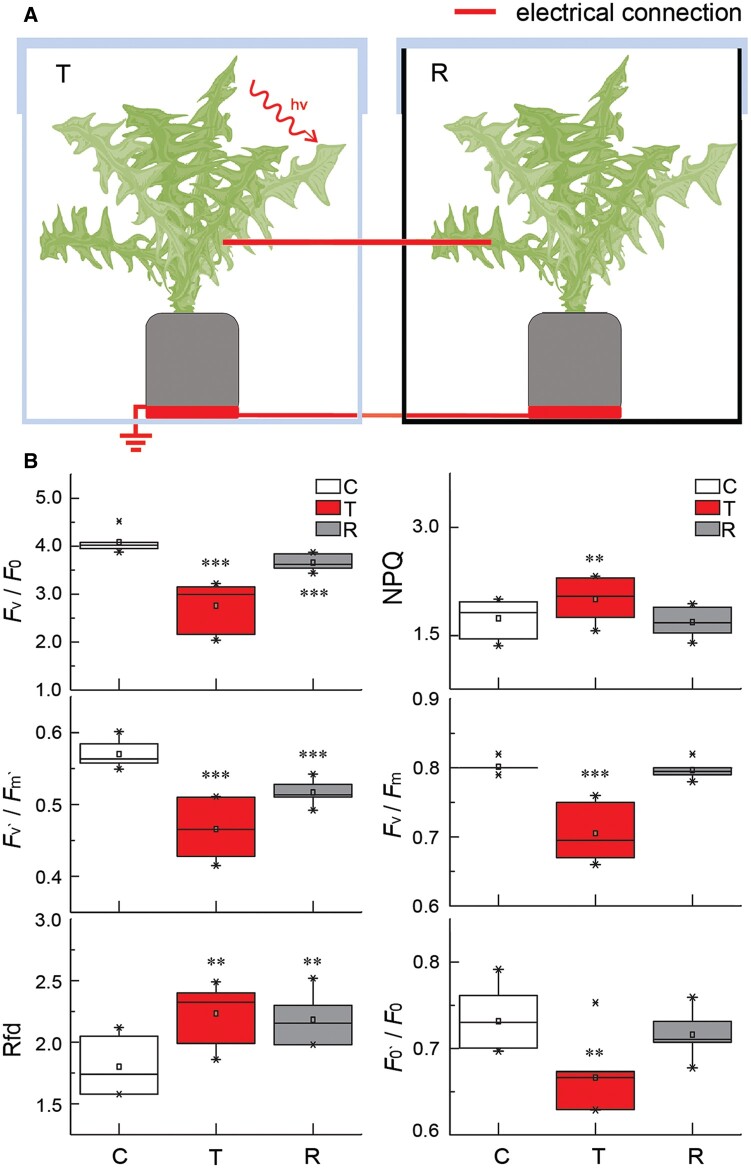
Changes in Chl *a* fluorescence in transmitter and receiver dandelion plants connected by a copper wire. A, Schematic diagram of the experiment presented in (B). Two different dandelion plants were placed on copper discs in separate plastic boxes, one of which was transparent (T, transmitter plant), while the other box had black walls (R, receiver plant). The plants and discs were coupled by copper wires threaded through the walls of the boxes. The ends of the wires coupling the plants were attached to the main vein of the leaves, and then the plants were grown for 2 days under standard laboratory conditions. Immediately prior to the experiment, the boxes were covered with lids and sealed. The T plant was stressed with a laser pointer light (2,000 μmol photons m^−2^ s^−1^, blue 450 nm), while the R plant was untreated. B, Quantification of Chl *a* fluorescence parameters in C, T, and R plants: potential photosynthetic activity (*F*_v_/*F*_0_), PSII maximum efficiency (*F*_v_′/*F*_m_′), Chl fluorescence decrease ratio (vitality index Rfd), NPQ, maximum quantum yield of PSII photochemistry in the dark-adapted state (*F*_v_/*F*_m_), fluorescence intensity ratio (*F*_0’_/*F*_0_). Control (C) are untreated plants before the experiment. ***P < 0.001; **P < 0.01; *P < 0.05, indicate significant differences between C and T as well as between C and R, as determined by Student’s *t* test. Data are shown as means � se (*n* = 10). The control experiment is presented in Supplemental Figure S10.

Interestingly, we also induced changes in Chl *a* fluorescence in the receiver plant by direct application of a DC (flows only in one direction) supplied through the wire from the transmitter plant (Figure�4 and Supplemental Figure S6), or from an external power source (Supplemental Figure S8). In contrast to DC, alternating current (AC) periodically reverses direction and changes in its magnitude continuously over time. When we exposed the transmitter plant to AC supplied as square waves (1V, 4H) from an external power source, we did not observe any changes in the dynamics of NPQ between control (untreated with AC) and transmitter (treated with AC) plants (Supplemental Figure S9). As a positive control for these experiments, we used thigmonastic movements in the sensitive plant *Mimosa pudica* L., which exhibits fast responses to electrical stimuli. Again, the responses were only induced in the DC system, when a heat-injured dandelion plant (transmitter, injury treatment of 1 and 5 s) transduced an electrical current through a metal wire and induced a response in the untreated mimosa plant (receiver) (Supplemental Movies S3and S4). Similarly, we induced a thigmonastic response in a mimosa plant when a heat-injured dandelion plant (primary transmitter) touched another dandelion plant (secondary transmitter), and the secondary transmitter was connected to untreated mimosa plant (receiver) through a metal wire (Supplemental Movie S5). We failed to induce a response in mimosa leaves in the systems consisting of (1) a dandelion plant treated with LaCl_3_ and then heat-injured with a metal stick (Supplemental Movie S6); (2) a dandelion plant (transmitter) connected to an untreated mimosa plant (receiver) through a metal wire, when the dandelion plant was touched with an unheated wooden stick, an unheated plastic stick (Supplemental Movie S7), or with a finger in a rubber glove (Supplemental Movie S8); or (3) a mimosa plant (receiver) treated with 0–50 V AC (Supplemental Movie S9). The sole exception was an induced mimosa response after treatment with AC exceeding 70 V, which represented a level of stress comparable to direct transmitter treatment (Supplemental Movies S9 and S10). Because ROS signals are not transmitted through the metal wire, we concluded that ESs are likely the main conduits mediating above-ground plant communication.

### Signal transduction and communication between a serially connected network of plants grown under field conditions

To examine the extent of plant-to-plant ES transmission and its possible function in nature, we measured changes in NPQ in plants arranged in a two-chain system under field conditions (Figure�5). In this experimental system, the plants touched each other and were sprayed with water prior to the application of stimuli and measurements. We measured significantly (*P* ≤ 0.01) higher mean NPQ values for the transmitter, primary receiver, and secondary receiver plants, compared to controls 30 min after point injury (Figure�5B). Importantly, 60 min after the initial treatment, NPQ remained at the same high level in R1, while NPQ further increased in R2 relative to the, indicating that the response develops in the receivers over time (Figure�5, B and C). This increase in NPQ was accompanied by a decrease in ΦPSII and qP values (Figure�5, D–G). These results demonstrate that aboveground plant-to-plant communication can occur within a network of plants touching each other.

**Figure 5:**
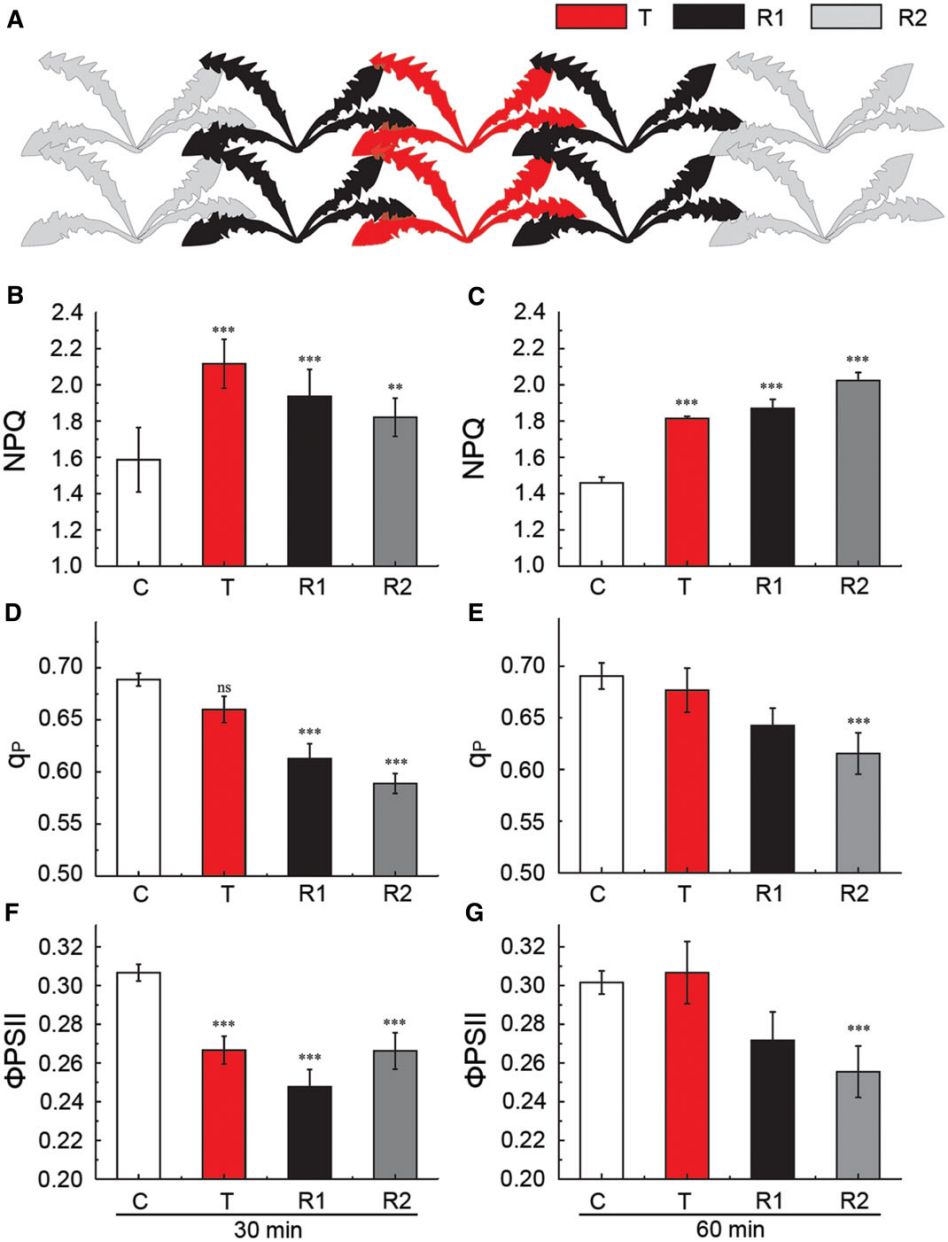
Changes in Chl *a* fluorescence in a chain of connected dandelion plants under field conditions. A, Schematic diagram of the experiments presented in B–G. Ten dandelion plants arranged in a two-chain system touching array were grown in one pot under laboratory conditions, before being moved to the field and acclimated for 1 week. Before the experiment, leaves were sprayed with water to ensure continuous contact between injured transmitter (T plants) and untreated receivers (R1 plants, primary receivers in direct contact with T; R2 plants, secondary receivers in direct contact with R1). B–G, Quantification of Chl *a* fluorescence parameters: NPQ, q_p_, and ΦPSII. Control (C) indicates uninjured T, R1, and R2 plants. ***P < 0.001; **P < 0.01 indicate significant differences between C and T as well as between C and R, as determined by Student’s *t* test; ns, not significant. Data are shown as means � se (C, *n* = 30; T, *n* = 6; R1, *n* = 12; and R2, *n* = 10). B, D, and F, Parameters measured at 30 min following injury of T. C, E, and G, Parameters measured at 60 min following injury of T.

### Induction of markers for SAA following aboveground plant-to-plant signal transduction and communication

In the experimental system allowing one-way signal transduction between plants (Figure�4), two parameters that might act as SAA indicators, that is, NPQ and H_2_O_2_ (Szechyńska-Hebda et al., 2010; Gilroy et al., 2016; Białasek et al., 2017), showed altered levels in the transmitter plant following injury and in the receiver plant as a result of plant-to-plant signaling (Figure�4 and Table�1, top panel). The higher values of H_2_O_2_ were accompanied by a close to 30- and 8-fold increase in superoxide dismutase (SOD) activity in the transmitter and receiver plant, respectively. While catalase (CAT) activity dropped by 10% in transmitter plants and by 33% in receiver plants, relative to the controls. Injury of the transmitter leaf increased jasmonic acid (JA) and abscisic acid (ABA) contents about 3-fold and 4.5-fold, respectively, but auxin levels dropped by about 36%; we also observed a 52% increase in salicylic acid (SA) contents specifically in the receiver plant. To further study responses related to acquired acclimation in receiver plants, we measured maximal PSII efficiency (*F*_v_/*F*_m_; a Chl *a* fluorescence parameter that indicates the level of photoinhibition; Baker, 2008) using the signal transduction system (Supplemental Figure S10), following excess light (EL) treatment (Table�1, bottom panel and Supplemental Figure S10). EL resulted in a significant (13%, *P* < 0.001) decrease of *F*_v_/*F*_m_ in the receiver of a pair of touching plants. However, *F*_v_/*F*_m_ only rose by a nonsignificant 3% after EL in a receiver plant that had previously received a signal from a heat-injured transmitter plant (Table�1), and in systemic leaves of the transmitter and receiver plants following local stress application (Supplemental Figure S10). Other parameters, like q_P_ and ΦPSII, were also higher in the receiver plant treated with EL, following earlier heat injury (in comparison to a receiver plant treated with EL only; Table�1). This finding indicated that EL induces SAA in the transmitter plant, as well as NAA in the receiver plant, as a result of ES and ES-induced ROS and electric signaling between plants.

**Table 1:** Changes in SAA markers and induction of NAA

SAA Marker	Unit	Control T		Injured T	Control R	R
H_2_O_2_	nmol g^−1^ FW	52.85 � 16.54		152.31 � 21.14 ^***^	56.74 � 16.24	71.34 � 32.51^*^
Total SOD activity	U mg^−1^ protein	0.85 � 0.75		25.24 � 7.58^***^	0.77 � 0.50	6.27 � 4.25^***^
Total CAT activity	�mol H_2_O_2_ mg^−1^ protein min^−1^	16.82 � 2.21		15.21 � 5.42	18.45 � 2.65	12.27 � 3.64
Total peroxidase activity	ΔA mg^−1^ protein min^−1^	0.72 � 1.10		2.11 � 0.54^*^	0.68 � 0.21	0.52 � 0.44
SA	�g g^−1^ FW	7.74 � 5.21		9.21 � 1.78	7.11 � 0.26	10.81 � 3.84^*^
JA	�g g^−1^ FW	95.73 � 34.24		300.18 � 29.21^***^	93.81 � 25.79	82.24 � 11.86
ABA	�g g^−1^ FW	0.71 � 0.11		3.22 � 1.18^***^	0.69 � 0.13	0.71 � 0.53
Cytokinins (Z + DHZ + tZR)	pg g^−1^ FW	17.81 � 2.61		21.13 � 5.31	16.74 � 2.16	18.46 � 4.10
Auxins (IAA + 4-CI-IAA + IBA)	pg g^−1^ FW	70.48 � 13.12		45.51 � 11.20^*^	74.67 � 6.23	65.84 � 9.11
Gibberellins (GA_3_ + GA_6_ + GA_7_ + GA_9_ + GA_12_)	�g g^−1^ FW	18.91 � 6.21		25.47 � 5.45	19.12 � 4.25	20.75 � 4.35

NAA		Control T + EL		Injured T + EL	Control R + EL	R + EL
*F*_v_/*F*_m_		0.662 � 0.033	0.686 � 0.019		0.660 � 0.030	0.746 � 0.020^***^
NPQ		0.992 � 0.099	0.954 � 0.090		0.992 � 0.099	1.002 � 0.084
q_P_		0.684 � 0.011	0.700 � 0.006^***^		0.684 � 0.011	0.732 � 0.006^***^
ΦPSII		0.453 � 0.022	0.480 � 0.014^**^		0.453 � 0.022	0.546 � 0.016^***^

*Notes*: The SAA experimental system consisting of dandelion plant pairs (transmitter T and receiver R) was designed according to Figure�4A and Supplemental Figure S7A. SAA parameters were measured before and after injury of T plants (Control T and Injured T) and in connected R plants (Control R and R). The NAA experimental system with pairs of Arabidopsis plants was designed according to Supplemental Figure S10. Parameters of Chl *a* fluorescence were measured in: uninjured T treated with EL (2,000 μmol photons m^−2^ s^−1^) for 30 min (Control T + EL); T, treated with EL after heat injury (Injured T + EL); R connected to “Control T+EL” plant (Control R + EL), R connected to “Injured T+EL” plant (R + EL).

*** Asterisks (for *P* < 0.001,

** for *P* < 0.01,

* for *P* < 0.05) indicate significant differences relative to control samples, as determined by Student’s *t* test. Data represent means � se (*n* = 12). FW, fresh weight.

### Aboveground plant-to-plant communication in Arabidopsis

We turned to Arabidopsis as an additional plant species, which can live within a plant community in the wild, to study plant-to-plant signal transduction at the molecular level. We first tested signal transduction between two leaves belonging to two different Arabidopsis plants (transmitter and receiver) in contact via touching following spraying the plants with water to induce high relative humidity and leaf-to-leaf conductivity. We determined that the pattern of aboveground plant-to-plant NPQ, temperature, and ES communication for Arabidopsis is similar to that seen with dandelion (Supplemental Figure S11). Changes in surface potential appeared to be dependent on the photosynthetic protein PsbS (an important component in dissipating EL energy via its regulation of NPQ), as demonstrated with the Arabidopsis *npq4-1* mutant, which lacks PsbS, and with a transgenic line overexpressing *PsbS* (*oePsbS*) (Supplemental Figure S12). We also observed the transduction of the ES between plants of different species, that is from an injured Arabidopsis transmitter to an untreated dandelion receiver (Supplemental Figure S13).

We investigated whether ES-induced changes in gene expression detected for two neighboring plants (Figure�6). For these experiments, we employed reporter constructs whereby the firefly luciferase (*LUC*) reporter gene is driven by the promoter regions of the *ZINC FINGER OF ARABIDOPSIS THALIANA 12* (*ZAT12*) or the *ASCORBATE PEROXIDASE 2* (*APX2*) promoter, and measured luminescence from control, transmitter, and receiver plants. *ZAT12* is a transcriptional repressor involved in light, temperature, salinity, wounding, biotic, and oxidative (H_2_O_2_) stress responses (Davletova et al., 2005). We detected higher LUC activity derived from the *ZAT12pro:LUC* reporter in both transmitter and receiver plants, when their leaves touched each other, after treating the transmitter plant only with light or wounding (Figure�6 and Supplemental Figure S14). Similarly, the *APX2pro:LUC* reporter, a marker of systemic oxidative stress signaling and SAA (Karpinski et al., 1999; Szechyńska-Hebda et al., 2010) produced higher LUC activity even if the signal was transduced between plants connected with a metal wire (Supplemental Figure S15).

**Figure 6:**
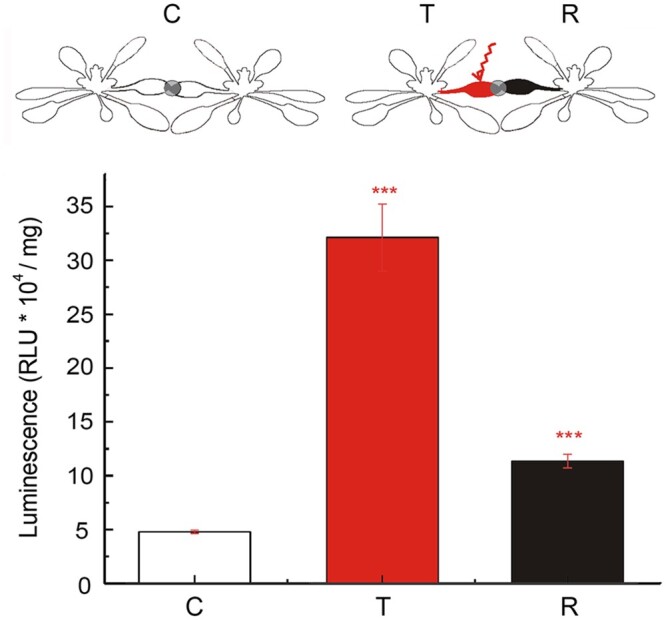
Induction of Arabidopsis *ZAT12* transcriptional response in transmitter and receiver plants. The experimental set consisted of a pair of Arabidopsis transgenic plants harboring the *ZAT12pro:LUC* transgene, whereby the transcription of the *LUC* reporter gene is under the control of the *ZAT12* promoter. The transmitter (T) plant was injured with a laser pointer light (2,000 μmol photons m^−2^ s^−1^), the receiver (R) plant was untreated, and both T and R leaves were connected to each other; the control (C) plant system consisted of two plants connected but untreated with EL. ***P < 0.001 indicate significant differences between C and T as well as between C and R, as determined by Student’s *t* test. Data are shown as means � se (*n* = 10).

We next tested the contribution of RESPIRATORY BURST OXIDASE HOMOLOGUE D (RBOHD), GLUTAMATE RECEPTOR-LIKEs (GLR3.3 and GLR3.6), and MECHANOSENSITIVE CHANNEL OF SMALL CONDUCTANCE-LIKE 10 (MSL10) to signal transduction. To this end, we employed the *rbohD*, *glr3.3 glr3.6*, and *msl10* mutants (Fichman et al., 2021) as mediators between transmitter and receiver (wild-type [WT]) plants in the system consisting of a chain of plants touching each other and sprayed with water. Under these conditions, we established that signal transduction from the injured transmitter to the receiver is blocked (Figure�7). In Arabidopsis, RBOHD is required for ROS production upon immune perception and abiotic stress cues (Castro et al., 2022). RBOHD is also required for the autopropagation of ROS signals (the so-called ROS wave), a mechanism essential for SAA within a plant (Miller et al., 2009; Fichman et al., 2021) and necessary for ROS transduction between different plants (Figure�7). GLRs have been implicated in alterations of both Ca^2+^ waves and surface potential from wounded to unwounded sections of the plant (Mousavi et al., 2013; Toyota et al., 2018). Ca^2+^ signals and damage-response membrane depolarizations are strongly attenuated in the *glr3.3 glr3.6* double mutant (Farmer et al., 2020). We detected the inhibition of ROS signaling when the *glr3.3 glr3.6* double mutant was used as the mediator in the chain of plants, as determined by H_2_DCFDA fluorescence (Figure�7). MSL10 has also been proposed as a regulator of ROS and long-distance electrical signaling (Veley et al., 2014; Farmer et al., 2020; Moe-Lange et al., 2021), and a *msl10* mediator plant blocked ROS signal transduction between transmitter and receiver plants (Figure�7). Taken together, our results link NAA to known regulators of Ca^2+^, ROS, and electric signaling, and demonstrate that NAA requires these important players.

**Figure 7:**
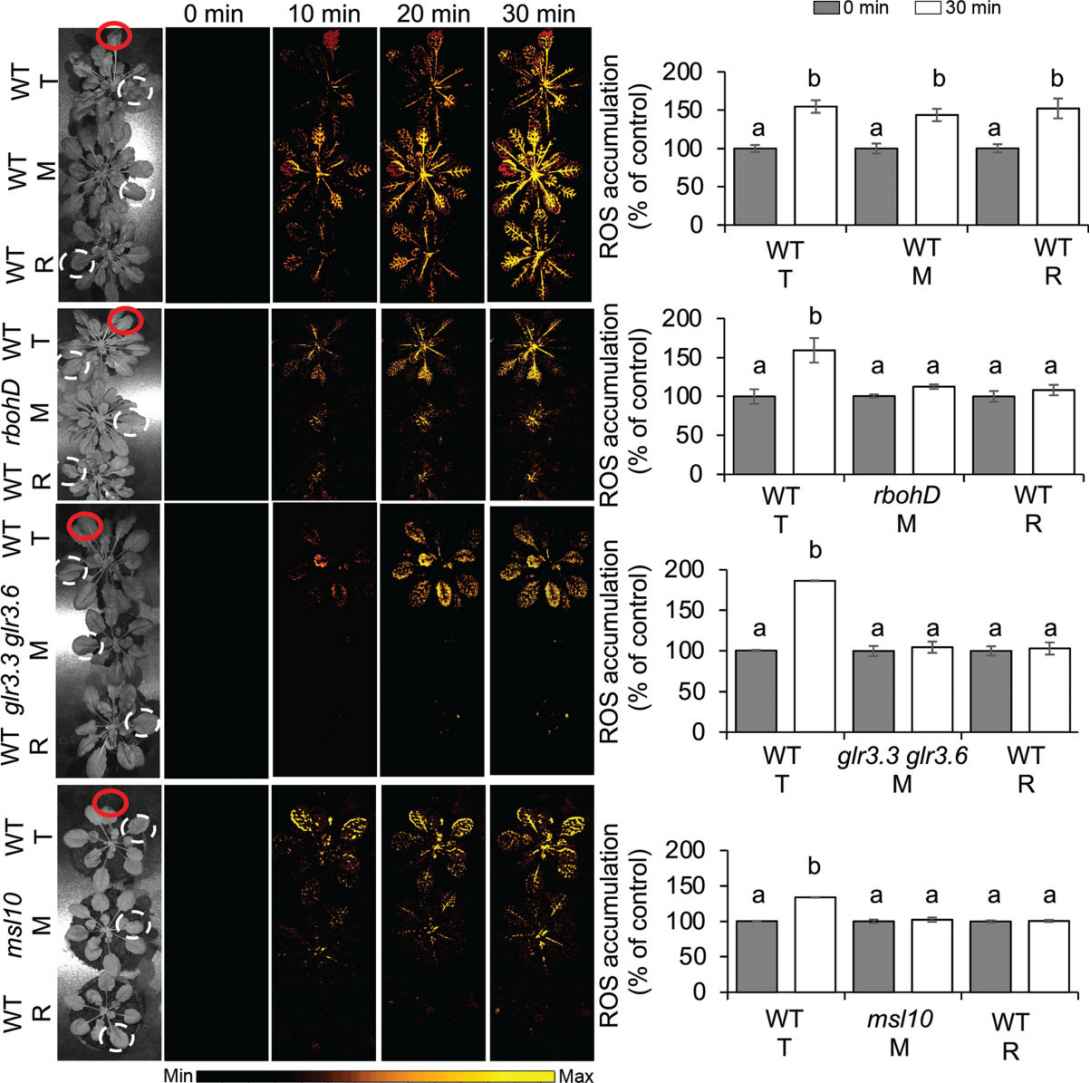
Inhibition of plant-to-plant ROS signaling in Arabidopsis rbohD, glr3.3 glr3.6, and msl10 mutants. A chain of plants touching each other consisting of a transmitter (T) and a receiver (R) WT (Col-0) plant were separated by a mediator plant (M): *rbohD*, *glr3.3 glr3.6*, or *msl10* mutant. Leaves of plants were connected by a simple touch and sprayed 60 min prior to experiment to ensure conductivity. One leaf of the T plant was touched with a heated metal wire (indicated by the red circle), while the other leaves were untreated. Pictures are representative examples of whole plant ROS imaging of triplets of plants as time lapse images at 0, 10, and 30 min after single-leaf injury. Color scales represent the range of values of the measured parameter. The corresponding graphs of ROS accumulation are results obtained at 0 min (gray bars) and 30 min postwounding (white bars). ROS were measured in the leaves marked with the white dashed circles. Different lowercase letters indicate significant differences (*P* < 0.05) as determined by ANOVA analysis followed by a Tukey’s test, ns, not significant. Data are shown as means � se (*n* = 8).

## Discussion

In Arabidopsis, a photoprotective mechanism has previously been discovered and described whereby a local leaf wounded or exposed to EL induces SAA in distal leaves (Karpinski et al., 1999; M�hlenbock et al., 2008; Pogson et al., 2008; Suzuki et al., 2013; Devireddy et al., 2018; Zandalinas et al., 2019). This process was shown to involve local and systemic changes in ES, NPQ, transcripts, metabolites, and phytohormone levels, stomata and other physiological responses, and to be mediated by systemic Ca^2+^, electric, hydraulic and ROS signals traveling from the stressed leaf to the entire plant (Szechyńska-Hebda et al., 2010; Gilroy et al., 2016; Mittler, 2017; Kollist et al., 2018; Fichman and Mittler, 2020, 2021; Tian et al., 2020; Sukhova and Sukhov, 2021). We hypothesized that plants not only communicate stress signals between different tissues and organs within the same plant to induce SAA, but that ES and ROS signals may also be transmitted from a wounded plant or a plant exposed to EL to nearby plants, if they touch each other.

Here, we show evidence that dandelion and Arabidopsis plants, which usually live in communities, can communicate with each other over long distances to induce acclimation responses as a network (community) of plants. We term this phenomenon “NAA.” An injured leaf on a particular plant can therefore generate systemic ES and ROS signals that are transmitted on the surface of the leaf to other plants. Although transmitted differently (i.e. on the leaf surface), ES is most likely similar to a slow wave potential (SWP) or system potential (SP) (Barres et al., 1976; Zimmermann et al., 2009; Kurenda et al., 2019; Farmer et al., 2020; G�recka et al., 2020; Sukhova and Sukhov, 2021) that are recorded extracellularly or intracellularly (the latter with reversed amplitude; Szechyńska-Hebda et al., 2010). Similarly to the SWP induced by wounding of Arabidopsis leaves (Farmer et al., 2020), the ES presented here: (1) began with a small (several microvolts) and transient membrane hyperpolarization, and some pulses of depolarization with a period of ∼50 s occurred within the slow repolarization phase (Figure�2, F–H); 2) moved within different areas of the same leaf (Figure�2F), from organ to organ (Figure�2G), and from plant to plant (Figure�2H). However, ES weakened with increasing distance from the site of injury (Figure�2, F–H). Similarly to SP that was investigated with microelectrodes positioned in substomatal cavities of both a dicot (fava bean, *Vicia faba*) and a monocot (barley, *Hordeum vulgare*) plant (Zimmermann et al., 2009), the ES measured here (3) modulated its amplitude (interdependent ion fluxes), from which the plant may be able to gain information about the nature and intensity of the injury; (4) needs to be to some extent a self-propagating signal, as the ES did not disappear completely with distance, but only weakened in amplitude (Figure�2, F–H), which was dependent on H^+^ pump activity changes (Zimmermann et al., 2009).

ESs transmitted internally through the vascular system, that is, through bundle sheath cells, or adjacent parenchyma cells, with a velocity of ∼0.5–5 mm s^−1^, can induce a range of systemic molecular, biochemical and physiological acclimatory responses in distant organs of the same plant (Szechyńska-Hebda et al., 2010; Salvador-Recatal� et al., 2014; Gilroy et al., 2016; Nguyen et al., 2018). In our experiments, ES propagated on the leaf surface with a velocity of ∼5 mm s^−1^ between different plants, or within a network of plants. This inter-plant ES was accompanied by specific physiological changes in Chl *a* fluorescence (NPQ, ΦPSII, qP, *F*_v_/*F*_m_, *F*_v_/*F*_o_, and *F*_v_′/*F*_m_′), biochemical changes in phytohormones (SA, JA, SA, and ABA) and ROS levels, and molecular alterations in gene expression (*APX2* and *ZAT12* induction), between transmitter and receiver plants. All parameters are robust markers for SAA induction (Karpinski et al., 1999; Pogson et al., 2008; Szechyńska-Hebda et al., 2010; Toyota et al., 2018; Fichman and Mittler, 2021), and here we provide evidence that they can also serve as NAA markers (Figure�8).

**Figure 8:**
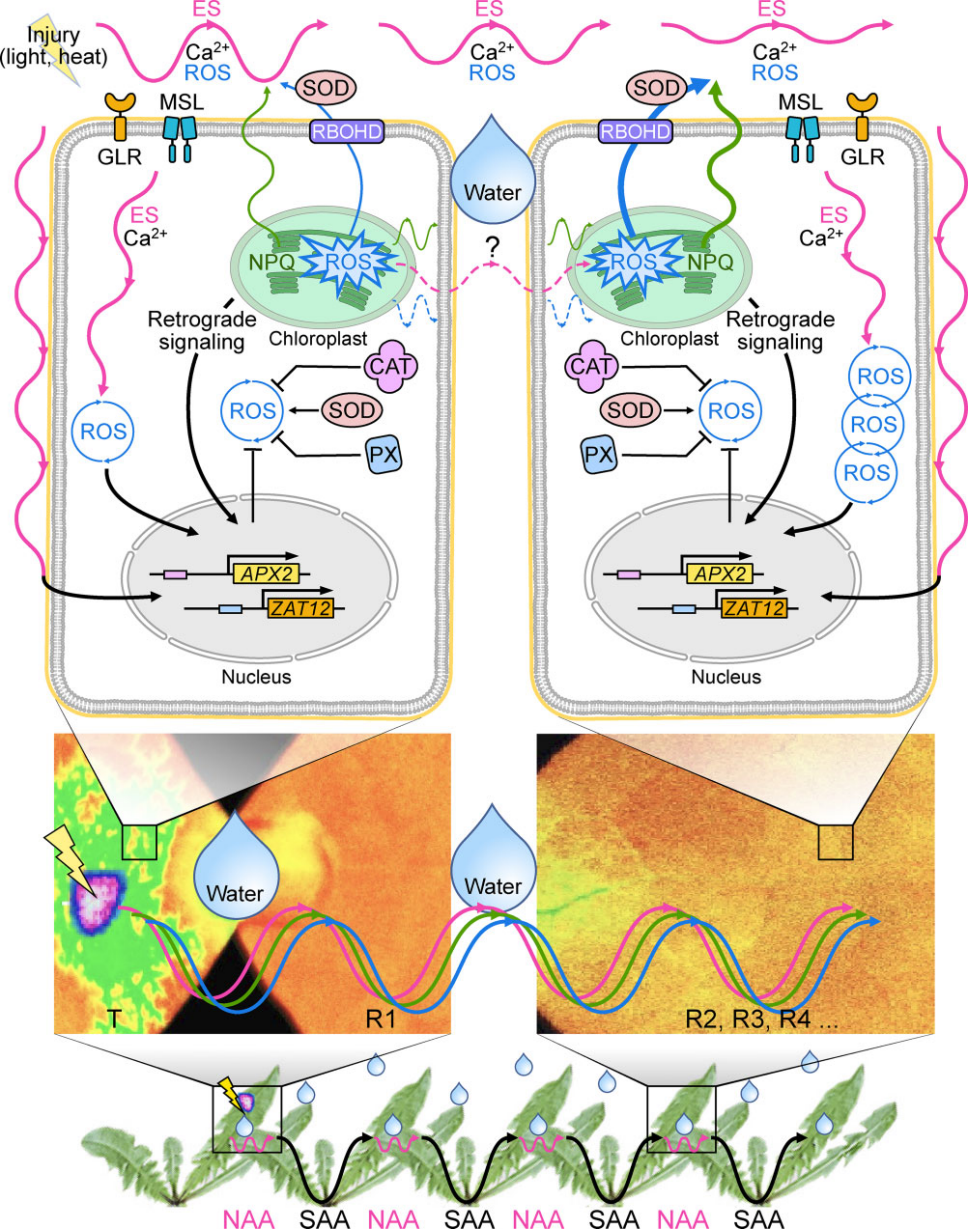
A model summarizing NAA responses in two different plants. Plants that live in a community, like dandelion or Arabidopsis, can use ES to communicate danger between each other and induce SAA within one plant and NAA between plants. Leaves belonging to two different plants (transmitter and receiver) need to be connected by a simple touch and electrical conductivity is required (e.g. high relative humidity, represented by a drop of water). Direct ES transmission (aboveground, on the leaf surface) between an injured (lightning arrow) transmitter (left cell) and an unstressed receiver plant (right cell) is most likely similar to a SWP or SP. ES has a modulated amplitude (interdependent ion fluxes) and drives spatiotemporal changes in energy quenching (NPQ), the subsequent induction of the ROS wave, and retrograde signaling (RS) in both transmitter and receiver plants. ROS wave propagation depends on the specific regulation by RBOHD, SOD, and CAT activities. Additionally, GRLs and MSL10 are implicated in alterations of ROS, Ca^2+^, and ES waves. The autopropagation of ROS can occur in the receiver cells. ES, the ROS wave, and RS induce gene expression changes in both in transmitter and receiver plants, such as for example *ZAT12* and *APX2*, markers of systemic signaling. Solid lines, ES-dependent signaling, ROS-dependent signaling; NPQ-dependent signaling; dashed line, hypothetical ES induced by current provided through a metal wire.

LaCl_3_, a Ca^2+^ antagonist that can be used as an inhibitor of Ca^2+^-dependent signaling, prevents ES propagation, systemic changes in PSII, systemic stomatal responses, and induction of acclimatory responses in directly exposed leaves, as well as in systemic leaves undergoing SAA (Szechyńska-Hebda et al., 2010; Białasek et al., 2017; Devireddy et al., 2018). We also established here that LaCl_3_ blocks ROS transduction in leaves of different plants undergoing NAA (Figure�3), supporting the involvement of ion channels and Ca^2+^ signaling in such processes. Long-distance communication between plants also required the function of RBOHD, GLR3.3 and GLR3.6, and MSL10, demonstrating that key regulators of systemic electric, Ca^2+^, and ROS signaling are involved in mediating NAA (Figure�8). Intraorganismal ES detected at the surface of leaves have been reported to mediate changes in the status of ions, pH, sugars, ROS, and turgor; changes in extracellular electric potential, and electric field; or even the induction of microvibrations that can be sensed by mechano-sensor channels that alter Ca^2+^ levels (Pogson et al., 2008; Toyota et al., 2018; Tian et al., 2020).

The existence of NAA between touching plants is also strengthened by the finding that applying stress to only one leaf of a single plant resulted in acclimation responses of remote plants within the network (community), and surprisingly, ES could mediate plant-to-plant intercommunication of stress signals between the same or across species aboveground. In nature, different types of aboveground plant-to-plant contacts can be considered, as plants shade each other, touch each other constantly or intermittently (depending on wind), or grow as part of a dense population or community of plants. Under humid or wet conditions, as demonstrated by our work, plants can transmit ES or even ROS signals between one another. The communication between isolated transmitter and receiver plants connected only by a copper wire circuit is also remarkable and important for understanding the nature of ES. In this experimental system, significant changes in PSII function, as indicated by *F*_v_/*F*_0_, *F*_v_′/*F*_m_′, and Rfd, the signaling molecule H_2_O_2_, and the contents of phytohormones, like SA, cytokinins, auxins, and gibberellins, resulted from the electrical information being carried by ions on the surface of the transmitter plant, before the movement of free electrons within the metal wire connecting plants, and finally again by ions in the receiver plant. A DC from an injured plant to a different plant (of same or different species) inducing complex responses strongly indicates that such signals are an important mechanism of plant communication. Plant cells can detect a voltage gradient as low as 0.5 μV/m and a current density as little as 5 nA/cm^2^ (O’Brien et al., 2017; Tyler, 2017). In our experimental system, a living plant was the source of current (ion fluxes), which was handed over to another plant through a wire (free electrons), excluding the possible influence of volatiles, or root contacts, since the two plants were in separate pots and enclosed in sealed chambers. This finding is supported by results showing that plants connected by their leaves differ in their parameters of Chl *a* fluorescence from those plants that are connected with a wire.

ROS induction and NPQ changes in receiver plants connected to a transmitter plant by touching are similar to ROS- and NPQ-dependent SAA responses that are essential for individual plants (Szechyńska-Hebda et al., 2010; Suzuki et al., 2013; Gilroy et al., 2016; Białasek et al., 2017; Devireddy et al., 2018; Zandalinas et al., 2019; G�recka et al., 2020). Recently ES was linked to the function of a 22-kD protein of PSII (PsbS), a key protein for the quenching and dissipation of excessive energy through NPQ and heat and thus ROS homeostasis in plants (G�recka et al., 2020). Because systemic ROS waves are thought to propagate via the active production and accumulation of H_2_O_2_ in the apoplast, and H_2_O_2_ is a stable form of ROS, it is possible that H_2_O_2_ molecules generated by the transmitter plants at the point of injury are transferred and together with ES trigger NPQ responses in the receiver plant (Fryer et al., 2003; Mullineaux et al., 2006; Szechyńska-Hebda et al., 2010; Fichman and Mittler, 2021). This, however, cannot happen when two plants are connected by a copper wire.

NAA may further be considered as a part of a complex of collective dynamics and emergent distributed computation in plants ecosystems. Peak et al. (2004) proposed a cellular-automaton model that explains how a plant leaf regulates its uptake of carbon dioxide, photosynthesis, and loss of water vapor during stress. The whole plant can develop tolerance after one leaf undergoes local stress (heat, wound, EL); ES, ROS, NPQ, PsbS, Ca^2+^ ions, photo-redox retrograde signaling, and induced cell death, are involved in this phenomenon (SAA; M�hlenbock et al., 2008; Szechyńska-Hebda et al., 2010; G�recka et al., 2020). NPQ and PsbS were linked to stress memory; stress had primed (acclimated) the plant and allowed it to survive subsequent stresses, but only in the presence of functional PsbS (G�recka et al., 2020). Here, we considered mechanisms that allow plants to dynamically adjust photosynthesis during stress through amplification and integration of multiple signals in the entire plant community (network). NAA relied on the components of SAA signaling; however, ES played a pivotal role due to transduction on the plant surface. We emphasized also the role of GLRs, MSL10, and RBOHD, which can integrate Ca^2+^, ROS, photosynthesis, and electric signaling and regulate responses to abiotic (SAA, hypoxia, heat, and wounding) and biotic (systemic acquired resistance, pathogens) stress triggered by cell death (Figure�8). Although this mechanism and its components are complex, its existence has great ecophysiological significance. Coordination of responses at the level of photosynthesis or stomatal responses (Devireddy et al., 2018; Kollist et al., 2018; Devireddy et al., 2020) of an entire plant can now be considered as part of a network coordinating the photosynthetic activity of different plants grown as part of the same canopy. Taking into account that few leaves per plant, thousands of cells per leaf, several dozens of chloroplasts per cell, and thousands of PSII per chloroplast, are potentially involved, reveals the complexity of the SAA and NAA signaling and communication mechanisms involved. A new inter-plant PSII-mediated signaling may even be considered.

Electrical conductivity between two plants is required for plant-to-plant ES transmission. Humid and conductive contacts were provided in our experimental system by spraying plants with water, or with a drop of agarose and water. Under field conditions, rainwater or dew would be an excellent conducting medium enabling ES transmission between different plants and supporting a role for plant-to-plant signaling in stress warning within an ecosystem. Moreover, ES between plants could be a viable alternative to signaling via volatile molecules, which is limited under wet and high humidity conditions. It is, therefore, possible that two alternative signaling pathways (volatiles and ES) exist as separate but complementary means for plant-to-plant communication, to ensure a high communication efficiency under a variety of environmental conditions. ES transmitted aboveground can be an important signaling route in the case of dangers such as damage by insects, disease, animals, which induce mechanical wounding, water status, and photosynthesis changes.

Fundamental questions do remain. First, considering a Darwinian point of view, what is the evolutionary significance of NAA? Could it simply be a side effect of the internal signaling networks of each plant, or does it lead to an evolutionary advantage to the plant? Second, to what extent can ES carry specific information and determine the range of responses (physiological, biochemical, and molecular) to a given stress? Third, if plants have the capacity for a rapid and precise recognition of hazard identification for survival, should plants with shared interests (e.g. the same species or different species that benefit from each other) induce “honest” (helping) signals beneficial to protecting the entire community, while plants with conflicting interests (e.g. different species that compete with each other) provide “cheating” (damaging) or “no signals,” as there could be selfishness involved? Fourth, considering that a network of plants growing together could have a shared adversary (e.g. herbivorous insects), is it possible that plants evolved the capability to communicate “danger” with each other in a rapid manner (that primarily includes aboveground plant-to-plant ES)? Fifth, rather than considering the adaptive value of NAA for the transmitter plant, should the perspective of the receiver plant be considered? Surveillance of defense signals from neighboring plants could provide an obvious value to the receiver plant. Much like spying, plants can extend their ability to sense the environment, including membrane polarity and voltage changes of a neighboring leaf. Because plants cannot afford to stop metabolism, ROS or electrical signals, as these are internally needed, the state of a neighboring plant can be easily monitored, and plants may have therefore evolved to take advantage of these signals to survey their environment. Sixth, can (or should) plants now be included in the group of organisms capable of using ES as warning and communication signals between individuals (similar to electroreception by aquatic or amphibious animals like sharks, rays, bony fish, dolphins, or electrolocation and electrocommunication by monotremes, cockroaches, and bees)? Further studies are needed to answer these and many other questions essential for this important mode of signaling and NAA.

## Materials and methods

### Plant material and growth conditions

Plant materials used in this study were *T.* *officinale* (common dandelion) and Arabidopsis (*A.* *thaliana*) plants in the Columbia-0 (Col-0) accession (WT; NASC stock number: N1092, Col-0), *atrbohD* (Torres et al., 2002; TAIR germplasm: CS68747), *glr3.3 glr3.6* (*glr3.3-1*, [SALK_099757C] and *glr3.6-1*, [SAIL_291_D06]; Mousavi et al., 2013), *msl10-1* (Basu et al., 2020; TAIR germplasm: CS72415), *npq4-1* (Li et al., 2000; TAIR germplasm: CS66021), *oePsbS* (Li et al., 2002; G�recka et al., 2020), *ZAT12pro:LUC* (Miller et al., 2009), and *APX2pro:LUC* (Karpinski et al., 1999). Plants were grown in soil mixed with perlite (3:1) in a growth chamber (23�C, 8-h light/16-h dark photoperiod with a light intensity of 200 � 15 �mol photons m^−2^ s^−1^). About 12- to 15-week-old dandelion plants and 4-week-old Arabidopsis plants were used for all experiments.

Some experiments were designed in such a way to rule out volatile signals or signals between root systems of plants in the same pot (both already documented methods of inter-plant communication). Individual plants were placed for each experiment on metal plates, so that the roots of individual plants were separated from each other, while transparent boxes were used in one experiment.

For field experiments, 12-week-old plants grown in a growth chamber were moved to field conditions during summer (Warsaw, 52�09′38″ N, 21�02′52″ E, 15-h light/9-h dark, 20�C–28�C during the day/14�C–24�C at night, 40%–70% humidity, rain protection), and acclimated at least 1 week before experiments.

For one-way signal transduction experiments, plants were placed on copper-grounded plates, allowing a single leaf from each plant to touch the other. Pairs of leaves growing on two different plants (transmitter and receiver) were sprayed with water to induce high relative humidity and leaf-to-leaf conductivity, thus mimicking naturally occurring touch during or following rain. To ensure electrical conductivity between plants, plants were also connected with a drop of water or a drop of 0.5% (w/v) agarose (Millipore-Sigma, Burlington, MA, USA). The control samples consisted of pairs of plants touching each other under dry conditions. Leaves were injured by heat for ∼1–2 s with a 1-mm-thick metal (stainless steel) stick heated directly with the flame of a butane lighter (Bic type, 800�C–1,000�C, ∼50�CW) for ∼15 s (e.g. see Supplemental Movies S3 and S4). For control measurements, a metal stick at ambient temperature was used.

A wire-transduced signal experiment was designed to: (1) recognize if a signal such as a weak electric current can induce any responses in receiver plants after injury of the transmitter plant; (2) confirm if a foliar signal originating at the light-stimulated plant and transduced to the receiver leaf via copper wire can induce responses and markers related to the classical type of SAA; and (3) exclude volatile plant signaling from the transmitter to the receiver plant. The experiment was designed, as shown in Figure�3A, in such way that the transmitter plant was kept in an enclosed transparent box, while the receiver plant was in a different plastic box with black walls. Two days before the experiments, plants were placed on metal-grounded plates, with one copper wire connecting the metal plates to ensure electrical conductivity between plants, while another copper wire, with a diameter at the tip not exceeding 0.1 mm, was introduced into the main veins of the transmitter and receiver leaves. The estimated introduction depth was 2 mm. For stabilization, the wire was previously twisted around the leaf (without damaging it) and the tip was strengthened with a drop of agarose after introduction. The wires pierced the box walls and the holes were sealed up with silicon. Plants were acclimated before the experiment under low light conditions (23�C, 8-h light/16-h dark with a light intensity of 200 � 15 �mol photons m^−2^ s^−1^). The plants were treated with a laser light (450 nm, 2-mm spot, 1 h at 2,000 μmol photons m^−2^ s^−1^) applied through the transparent lid.

Signal transduction between serially-connected plants was measured for plants grown under field conditions to examine the extent of plant-to-plant canopy ES transmission and its possible function in nature. As illustrated in Figure�5A, ten plants were arranged in a two-chain system. They were grown in one pot along two rows, five plants each. Plants were placed on metal plates, so that the roots of individual plants were separated from each other. In contrast, leaves of 8-week-old plants touched each other. Plants were initially grown under laboratory conditions, before being moved to the field and acclimated for at least 1 week before the experiment. Just before the experiment, plants were sprayed with water to ensure continuous contact between plants that touched each other. The heat injury treatment was applied to central plants, as with the one-way system.

For the induction of acquired acclimation in receiver plants, two different plants were placed on copper discs. The plants touched each other via individual leaves. Plants were sprayed with water and kept under low light conditions for 30 min before they were heat-injured, followed by an additional 30 min in low light conditions. The plants were then treated for 1 h with EL (2,000 � 100 �mol photons m^−2^ s^−1^, white light supplied by light-emitting diode panels, Photon System Inst.). The transmission of injury-related signals from the transmitter (injured) plants to receiver (noninjured) plants was measured as changes in Chl *a* fluorescence parameters after next 30 min of dark conditions.

### Chl *a* fluorescence

Spatiotemporal measurements of Chl *a* fluorescence were conducted with an Imaging-Pam Mini fluorometer and ImagingWin software (Heinz Walz GmbH, Effeltrich, Germany). The imaged leaf area was set to 32 � 24 mm. Plants were kept in darkness for 20 min, then a blue actinic light (*λ*_max_ = 470 nm, 60 μmol photons m^−2^ s^−1^) was switched on for ∼30 min. To measure *F*_m_ and *F*_m0_, saturating pulses at 20-s intervals were applied (6,000 μmol photons m^−2^ s^−1^, duration 800 ms). After exposure to light, relaxation of NPQ in darkness was monitored. Maximum fluorescence was recorded every 30 s. Single exponential functions for relaxation of NPQ were fitted. Chl *a* fluorescence under field conditions was measured using a Chl fluorometer (FluorPen FP 100) equipped with a PIN photodiode detector, a 667–750 nm bandpass filter, and FluorPen version 1.0 software (Photon System Instruments, Brno, Czech Republic). The measured leaf area diameter was 5 mm to provide a standard window of the detachable leaf-clips. Prior to measurement the plants were kept in darkness (closed window of the leaf-clips) for 20 min, and then a blue actinic light (λ_max_ = 470 nm, 300 μmol photons m^−2^ s^−1^) was switched on. To measure *F*_m_ and *F*_0_, saturating pulses were applied (2,100 μmol photons m^−2^ s^−1^). NPQ, ΦPSII, and qP were measured and calculated by using the NPQ1 protocol predefined by the manufacturer.

NPQ (*F*_m_ – *F*_m0_/*F*_m0_), effective quantum yield of PSII (ΦPSII, [*F*_m0_– F]/*F*_m0_), and qP (*F*_m0_– *F*/*F*_m0_ –*F*_0_) were determined according to the manufacturer instruction. *F*_v_/*F*_0_ is a parameter that accounts for the simultaneous variations in *F*_m_ and *F*_0_ in determinations of the maximum quantum yield of PSII. *F*_v_′/*F*_m_′ provides an estimate of the maximum efficiency of PSII photochemistry at a given Photosynthetic Photon Flux Density (PPFD), which is the PSII operating efficiency if all the PSII centers are “open” (QA fully oxidized). Rfd (the Chl fluorescence decrease ratio) is defined as Chl fluorescence decrease *F*_d_ from maximum to steady state fluorescence *F*_s_; *F*_d_/*F*_s_ is determined by the Kautsky effect. *F*_0_′/*F*_0_ gives the ratio of: (1) the minimal level of fluorescence after far-red light (30 μmol photons m^−2^ s^−1^ at 720–730 nm for 4 s) that excites PSI preferentially, and thus oxidizes the plastoquinone and QA pools associated with PSII and (2) the minimal level of fluorescence, after exposure of a dark-adapted leaf to a weak modulated measuring beam (PPFD of ca. 0.1 μmol m^−2^ s^−1^).

### Transpiration

Fully developed leaves were cut off the plants and weighed. A hole was made in the cap of each Falcon tube. The leaf petiole was introduced in such a way, that the leaf was above the Falcon cap, while the petiole tip was inside the Falcon tube and immersed in tap water. The caps were then sealed with parafilm. Injured and control leaves were weighed together with Falcon tubes in 30-min intervals for 2 h. Leaf size was measured with the FluorCam 800MF after calibration with a fluorescent marker (Photon System Instruments, Czech Republic). The transpiration rate was expressed as the ratio of water loss (g H_2_O) per leaf area (cm^2^).

### Leaf temperature

Leaf temperature was measured using an FLIR T650sc IR camera with FLIR ResearchIR version 3.4 software (FLIR Systems, Wilsonville, OR, USA). To measure the foliar temperature, an emissivity of 0.95 and a frequency of one frame per second were used. Detached leaves were dark-adapted for 20 min. Light treatment was applied using a red LED panel (*λ*_max_ = 627 nm, Photon System Instruments, Czech Republic), and four intensities of light (375, 750, 1,500, and 3,000 μmol photons m^−2^ s^−1^), each for 2 min, separated by 1 min of darkness. Thermal images were taken with a frequency of one frame per second. The average temperature was measured at the circled areas above and below the injury. To describe the relationship between temperature and light intensity, a linear function was fitted (*R*^2^ > 0.98). The fitted models were compared using one-way analysis of variance (ANOVA).

### ROS imaging with H_2_DCFDA

Based on a previously described protocol (Fichman et al., 2019), imaging of whole-plant ROS levels was conducted 30 min after fumigation of plants with 50 μM H_2_DCFDA (excitation/emission 495 nm/517 nm; Millipore-Sigma, USA) buffered in 50 mM phosphate solution (pH 7.4). Plants were then treated and imaged for 2 h using the IVIS Lemina S5 platform (PerkinElmer, Waltham, MA, USA). For ROS signaling inhibition, a drop of 2 mM LaCl_3_ was placed on the touching leaves from the two plants 60 min prior to wounding (Devireddy et al., 2018). Images were taken every 30 s for 30 min. Accumulation of the signal was calculated using Living Images version 4.7.2 Software (PerkinElmer, USA).

### Electrical potential at the leaf surface

The term “electrical signaling” is used for extracellular changes in the potential, which represent the sum of the electrical activity of many cells, detected with voltage-sensitive glass microelectrodes. Silver electrodes (0.5 mm in diameter, World Precision Instruments, Sarasota, FL, USA) were cleaned with sandpaper, 70% (v/v) ethanol, rinsed in deionized water, and then chloridized with 30% (w/v) NaClO solution for 30 min (Ag/AgCl) and rinsed again with deionized water. Glass microelectrodes were prepared from aluminosilicate tubing with filament from borosilicate glass capillaries with filament (inner diameter of 0.64 mm, outer diameter 1.00 mm, length of 10 cm, Sutter Instrument) by heating and pulling with a PC-10 vertical micropipette puller (Narishige, Tokyo, Japan, heat level set to 24.8). The tip diameter was <0.5 μm. Microelectrodes were filled with 1 M KCl, and connected to a headstage (HS-9Ax0.1U, R0 = 100 MΩ; HS-9Ax1U R0 = 10 MΩ, Molecular Devices, San Jose, CA, USA) via the Ag/AgCl electrode. The tip potential was –5 to –15 mV, and the resistance was 5–15 mΩ. The tip resistance was ∼50 MΩ, the input resistance of the headstage was ∼10^13^ Ω. The leaves were connected with microelectrode by a three-axis micromanipulator (Narashige, Tokyo, Japan) via a drop (20 mL) of 1 mM KCl/0.5% (w/v) agarose to avoid direct contact with plant cells and damage the cuticle. The ground electrode (Ag/AgCl) was placed in the soil in which the plant was rooted. An Axon Axoclamp 900A Microelectrode Amplifier (Molecular Devices, USA) with Current clamp (I-Clamp) mode, for measuring voltage responses with I = 0 option, and pCLAMP10 software (Molecular Devices, USA) was used to record the electric potential (sampling frequency 0.5 kHz). Experiments were conducted in a Faraday cage at room temperature (22�C–25�C). Plants were watered well 1 day before experiments, positioned on a grounded copper plate, and sprayed with water to ensure electrical conductivity. After stabilization of all parameters, heat treatment was performed, while the time of injury was set as the beginning of record to normalize the electrical curves.

### Gene expression

LUC activity derived from the *ZAT12:LUC* and *APX1:LUC* transgenic lines was quantified in three leaves from each rosette as a proxy for *ZAT12* and *APX1* expression. Collected samples (∼5 mg) were ground in 0.5 mL lysis buffer (Promega kit). About 50 μL of the homogenate was placed under a luminometer tube (Berthold), and 50 μL of luciferin assay was added 10 s before the measurement.

### Biochemical analysis

Plant leaves homogenized in phosphate buffer (pH 7.0) with 0.1 mM ethylenediaminetetraacetic acid and 1% (w/v) polyvinylpyrrolidone were used for spectrophotometric (Perkin Elmer LS 50B, Norwalk, USA) analysis of hydrogen peroxide and antioxidant enzymes, as previously described (Szechyńska-Hebda et al., 2007, 2010). Hydrogen peroxide contents were determined with homovanillic acid and peroxidase. The absorbance was measured at 315 nm. SOD activity was determined with nitroblue tetrazolium (NBT), xanthine and xanthine oxidase. The absorbance at 560 nm was recorded for 2 min. SOD activity was calculated as the percentage inhibition of NBT reduction. CAT activity was measured based on the rate of H_2_O_2_ decomposition, which is proportional to the reduction in absorbance at 240 nm. Peroxidase activity (PX) was measured using p-phenylenediamine as the substrate. One unit of PX activity (ΔA) was defined as the change in absorbance at 460 nm per min and expressed in terms of units per mg of protein. Protein contents were determined with the Bradford assay, using bovine serum albumin as a standard.

For the extraction and quantification of phytohormones, lyophilized leaves were pulverized in a mixing mill (Retsch, Haan, Germany), and the dried powder was used for phytohormone analysis *(*Kojima et al., 2009). Briefly, samples were labeled with internal standards (Millipore-Sigma, USA), extracted twice using a mixture of methanol/water/formic acid (75:20:5, v/v/v), and purified on solid-phase extraction cartridges (BondElut Plexa PCX, 30 mg, 1 mm, Agilent Technologies, Santa Clara, CA, USA). The fraction containing SA, JA, ABA, auxins, and gibberellins was combined with the cytokinin fraction, evaporated under a stream of nitrogen, reconstituted in acetonitrile, filtered (0.22-μm nylon membrane), and analyzed by ultra-high-performance liquid chromatography (Agilent Infinity 1260, Agilent, Germany) and triple-quadrupole mass spectrometry (Agilent 6410, Agilent, USA). Separation was achieved on an Ascentis Express RP-Amide analytical column (2.7 μm, 2.1 mm � 75 mm; Millipore-Sigma, USA).

### Statistics and data analysis

All data analysis and statistics were performed in R (R Core Team, 2015). Values are presented as means and standard errors (se). *n* represents the number of independent plants used. Asterisks indicate the significance levels: *P ≤ 0.05; **P ≤ 0.01; ***P ≤ 0.001 relative to control samples, as determined with Student’s *t* tests. Different letters indicate significant differences, as determined with Tukey’s honestly significant difference (HSD) test. For multiple curves comparison and to allow presentation in one figure, a normalization of the results was performed to fit the starting point to 0 or another value, if applicable (without other data transformation).

## Accession numbers

Sequence data used in this study can be found in the Arabidopsis Information Resource (https://www.arabidopsis.org) under the following accession numbers: *RBOHD* (At5g47910); *GLR3.3-1* (At1g42540); *GLR3.6-1* (At3g51480); *MSL10* (At5g12080); *NPQ4* (At1g44575); *PsbS* (At1g44575); *ZAT12* (At5g59820); and *APX2* (At3g09640).

## Supplemental data

The following materials are available in the online version of this article.

**Supplemental Data Set S1.** Summary of statistical analyses.

**Supplemental Figure S1.** Systemic changes in temperature, NPQ, and transpiration of dandelion leaves following a heat injury.

**Supplemental Figure S2.** Plant-to-plant transmission of NPQ and ROS following a heat injury.

**Supplemental Figure S3.** Transmission of injury signal, as revealed from changes in NPQ, from transmitter to receiver dandelion plants.

**Supplemental Figure S4.** Changes in the efficiency of PSII (ΦPSII) and qP PSII of dandelion leaves following a heat injury.

**Supplemental Figure S5.** ES transmission between transmitter and receiver dandelion plants.

**Supplemental Figure S6.** Communication between two dandelion plants connected by a wire.

**Supplemental Figure S7.** Control setup for Figure�4.

**Supplemental Figure S8.** Spatiotemporal changes in NPQ and yield of PSII efficiency (Y(PSII)) after electrical treatment.

**Supplemental Figure S9.** Stimulation of dandelion leaves by AC.

**Supplemental Figure S10.** Acquired acclimation responses between transmitter and receiver plants.

**Supplemental Figure 11.** Physiological changes of Arabidopsis and dandelion leaves following light treatment.

**Supplemental Figure S12.** ES generation in Arabidopsis Col-0, *npq*, and *oePsbS* plants.

**Supplemental Figure S13.** ES generation in one Arabidopsis plant and ES signal transduction between one Arabidopsis plant and one dandelion plant after heat injury.

**Supplemental Figure S14.** SAA signal transduction between two leaves of one plant induces gene response.

**Supplemental Figure S15.** SAA signal transduction between two different plants induces gene response.

**Supplemental Table S1.** Mean value of NPQ, ΦPSII and qP as a function of distance from injury (5, 10, 15, 20, and 25 mm).

**Supplemental Table S2.** The dark relaxation of the NPQ.

**Supplemental Table S3.** ES characteristics.

**Supplemental Movie S1.** Turgor changes of the dandelion leaf toughed 3 times with heated metal wire.

**Supplemental Movie S2.** Signal transduction between dandelion plants under laboratory conditions.

**Supplemental Movie S3.** Signal transduction between plants of two different species after 1–2 s heat injuring treatment.

**Supplemental Movie S4.** Signal transduction between plants of two different species after 5 s heat injuring treatment.

**Supplemental Movie S5.** NAA signal transduction between three plants of two different species after 1 s of heat injuring treatment.

**Supplemental Movie S6.** Signal transduction between plants of two different species treated with LaCl_3_.

**Supplemental Movie S7.** Lack of signal transduction between plants of two different species toughed with unheated plastic stick, wood stick, and metal wire.

**Supplemental Movie S8.** Lack of signal transduction between plants of two different species toughed with hand in a rubber glove.

**Supplemental Movie S9.** Electrical stimulation with an AC (0–133 V AC).

**Supplemental Movie S10.** Electrical stimulation with an AC (∼240 V AC).

## Supplementary Material

koac150_Supplementary_Data

## Notes

S.M.K. formulated the hypothesis and conceived the research plan. S.M.K., M.S.-H., and R.M. supervised the research. M.S.-H., M.L., D.W., and Y.F. performed the experiments and analyzed the data. M.S.-H., M.L., S.M.K., and R.M. interpreted the results and wrote the manuscript.

The author responsible for distribution of materials integral to the findings presented in this article in accordance with the policy described in the Instructions for Authors (https://academic.oup.com/plcell) is: Stanislaw M. Karpiński (stanislaw_karpinski@sggw.edu.pl).
