# Supplementary material for: Aboveground plant-to-plant electrical signaling mediates network acquired acclimation
Source: Plant Cell. 2022 May 20;34(8):3047–65. doi: 10.1093/plcell/koac150 (PMC9338792; doi:10.1093/plcell/koac150)
Supplement: koac150_Supplementary_Data [file koac150_supplementary_data.zip › koac150-suppl_data/TPC20221RA00825R3 Suppl Figures and Tables.pdf]

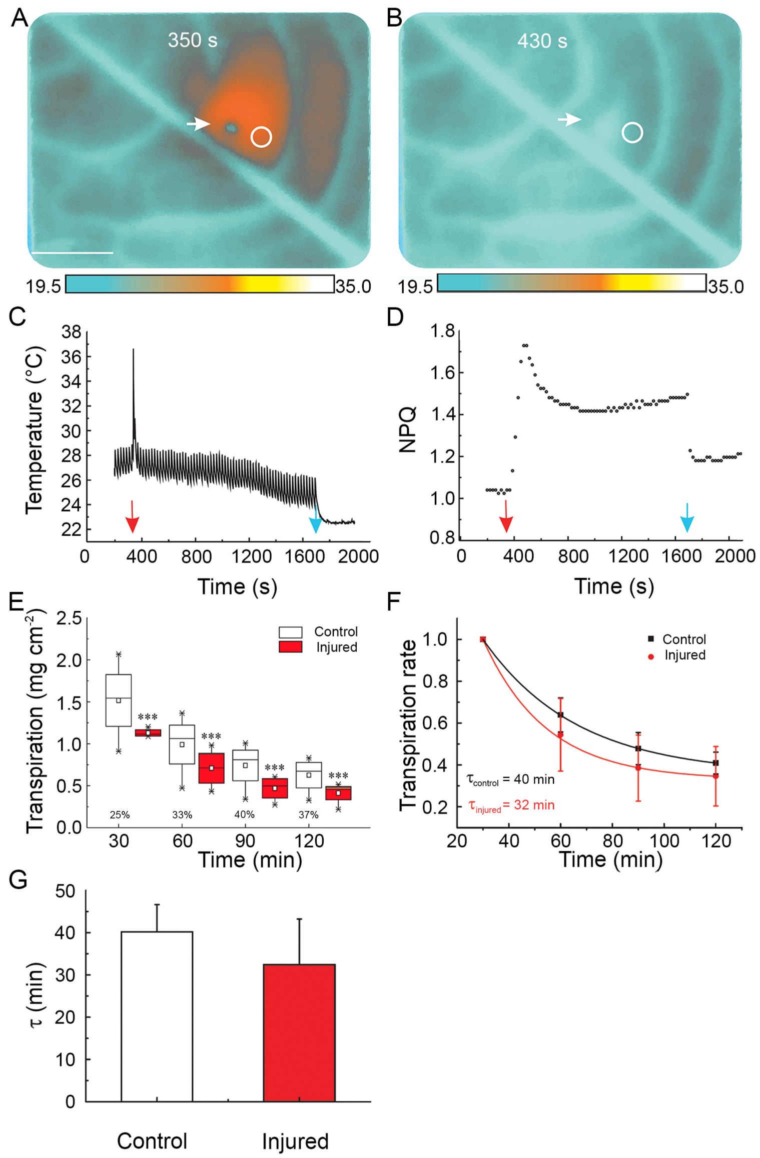

Supplemental Figure S1. Systemic changes in temperature, nonphotochemical quenching (NPQ), and transpiration of dandelion leaves following a heat injury. (A-B) Representative imaging of spatiotemporal changes in foliar temperature, at 350 s, and 430 s of the recording in Figure 1C. The white circles indicate the analyzed areas. Color scales represent the range of values of the measured parameter. Scale bar = 1 cm. (C-D) Temperature and NPQ kinetics. (A-D) Data recorded over 2,000 s (n=5). Injury (2 s) was applied at 350 s. White and red arrows indicate the injury with the metal wire. (C-D) blue arrows indicate the time when actinic light was turned off. (E) Transpiration of uninjured control and heat-injured plant. Percentages indicate the difference between control and injured leaves; ***, $p < 0.001$ indicate significant differences between control and injured leaves, as determined by Student's t-test (n=4). (F) Transpiration rate as a single exponential function (r^2> 0.98) in time for control and heat-injured plants. Comparison of the fitted curves reveals a lower decay time for heatinjured ($t_{iinjured}$= 32±11 s) than control ($t_{control}$= 40±6 s) plants. (G) Mean decay time for control and injured plants. ns, not significant. (E-G) Dandelion leaves were cut off and placed in a tube filled with water, measurements were carried out over 120 min in 30-min intervals. Data are shown as mean ± SE (n=4).
Supports Figure 1.

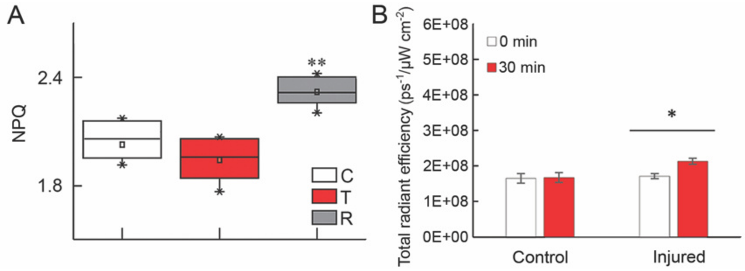

Supplemental Figure S2. Plant-to-plant transmission of NPQ and ROS following a heat injury. The leaves of two different dandelion plants were connected by a simple touch following spraying with water to ensure conductivity. A pair of transmitter (T) and receiver (R) leaves was analyzed, with the T leaf touched (2 s) with a heated metal wire; in a pair of control (C) leaves, one leaf was touched with an unheated metal wire. (A) NPQ changes (n=15). (B) Quantification of ROS fluorescence at 0 min and 30 min after wounding (n=6). (A-B) **, $p < 0.01$; *, $p < 0.05$ indicate a significant difference between C and R plant, as determined by a Student's t-test. Data are shown as mean ± SE.
Supports Figure 2.

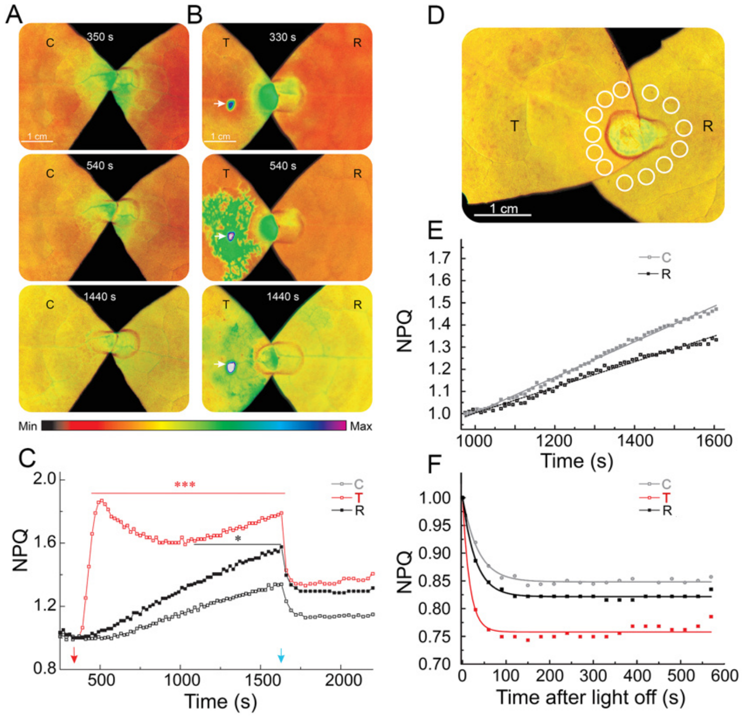

Supplemental Figure S3. Transmission of injury signal, as revealed from NPQ changes, from transmitter to receiver dandelion plants. The leaves of two different dandelion plants were connected by a drop of agarose to ensure contact. Plants were placed on one copper plate; however, the roots of the individual plants were separated from each other. Plants were watered well to obtain a closed-circuit system. Heat injury was applied at 330 s**.** Actinic light was applied over 1,630 s**,** and then the light was switched off (blue arrow) for the next 300 s. (A) Representative images of NPQ at 330, 540, and 1440 s for a control pair of plants, one of which (C) was touched with an unheated metal wire after injury. (B) Representative images of NPQ of a pair of plants. The transmitter (T) was touched with a heated metal wire and the receiver (R) was untreated. (C) NPQ kinetics for whole leaf area of C (n=50), T (n=33), R (n=38) leaves (normalized records). ***, $p < 0.001$; * $p < 0.05$ indicate significant differences between C and T (red asterisks) or between C and R (black asterisk), as determined by Student's t-test; pairwise comparisons at the same time point were performed; a longer line indicates that more values were significantly different between C *vs* T curves (red line) and C *vs* T curves (black line). (D) NPQ analyzed area for Supplemental Figure S3, E and F, indicated by white circles. (E) The linear function (r^2>0.98, slope=$6.58 \cdot 10^{-5}$ and 5.67 10^{-54} for R and T, respectively) fitted to average NPQ values measured over time from 1,000 s to 1.570 s (corresponding to Supplemental Figure S3C) for T (n=36) and R (n=30). (F) Dark relaxation of NPQ recorded after turning the actinic light off for T (n=39), R (n=35), and C (n= 31). A single exponential decay model was fitted ($r^2 \geq 0.98$).

Supports Figure 2.

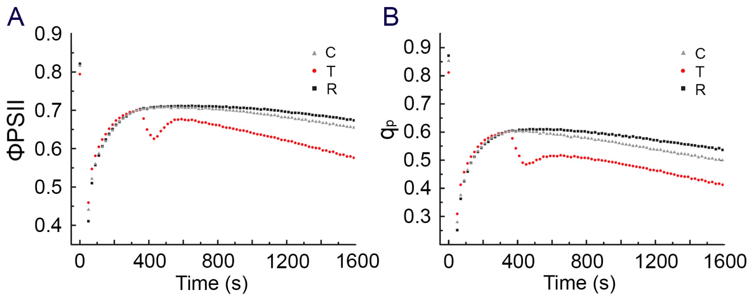

Supplemental Figure S4. Changes in the efficiency of PSII (FPSII) and photochemical quenching of PSII (q_P) of dandelion leaves following a heat injury. The leaves of two different dandelion plants were connected by a drop of agarose to ensure contact. Plants were placed on one copper plate; however, the roots of the individual plants were separated from each other. Plants were watered well to obtain a closed-circuit system. Heat injury was applied at 330 s**.** Actinic light was applied over 1,630 s**,** and then the light was switched off for the next 300 s (as in Supplemental Figure S3). Changes in (A) FPSII and (B) q_P for control (C, n=33), transmitter (T, n=22), receiver (R, n=26) plants (normalized records).
Supports Figure 2.

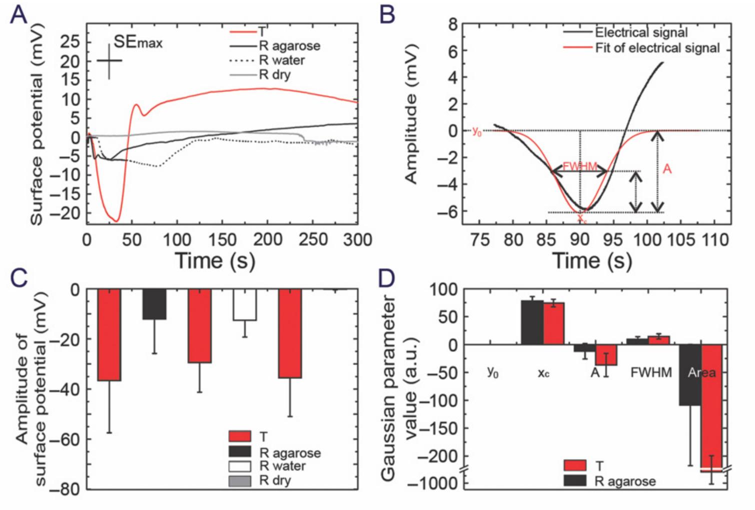

Supplemental Figure S5. ES transmission between transmitter and receiver dandelion plants. To compare signal propagation between leaves of different dandelion plants *i.e.*, transmitter (T) and receiver (R), various types of contact between leaves were tested: a drop of agarose (T-agarose-R), a drop of water (T-water-R), and direct contact without any connector (T-dry-R). (A) Electrical signals (ES) recorded at the surface of the leaf for T (red solid curve), as well as R connected to T by a drop of agarose (black solid curve), a drop of water (black dotted curve), and dry connection (gray solid curve) (normalized records). Maximal SE is indicated for the x-axis (time) and y-axis (surface potential) by the length of crossed error bars. (B) The Gaussian function fitted to the ES values generated after injury in the T-agarose-R system, where y_0 for T and R is fixed to 0; $R^2 > 0.8$; and x_c describes the time at which ES reaches a maximum amplitude. (C) Mean amplitudes of signal transduction for T-agarose-R, T-water-R, T-dry-R systems. (D) Parameters of the function presented in Supplemental Figure S5B for T-agarose-R systems. Note: Although heat injury was not standardized (small differences existed in the time of treatment and injury area), the average ES amplitude for the transmitter was between –25 and –35 mV in each subsequent experiment. The amplitude for R in both systems, T-agarose-R, T-water-R, was from –5 to –10 mV. No changes in electrical potential on the R surface were recorded when the plants were in dry contact (the amplitude was close to 0 mV). (B, D) Offset (y_0), center (x_c), maximum amplitude (A), full width at half maximum (FWHM), and integral area under the curve.

Supports Figure 2, F-H.

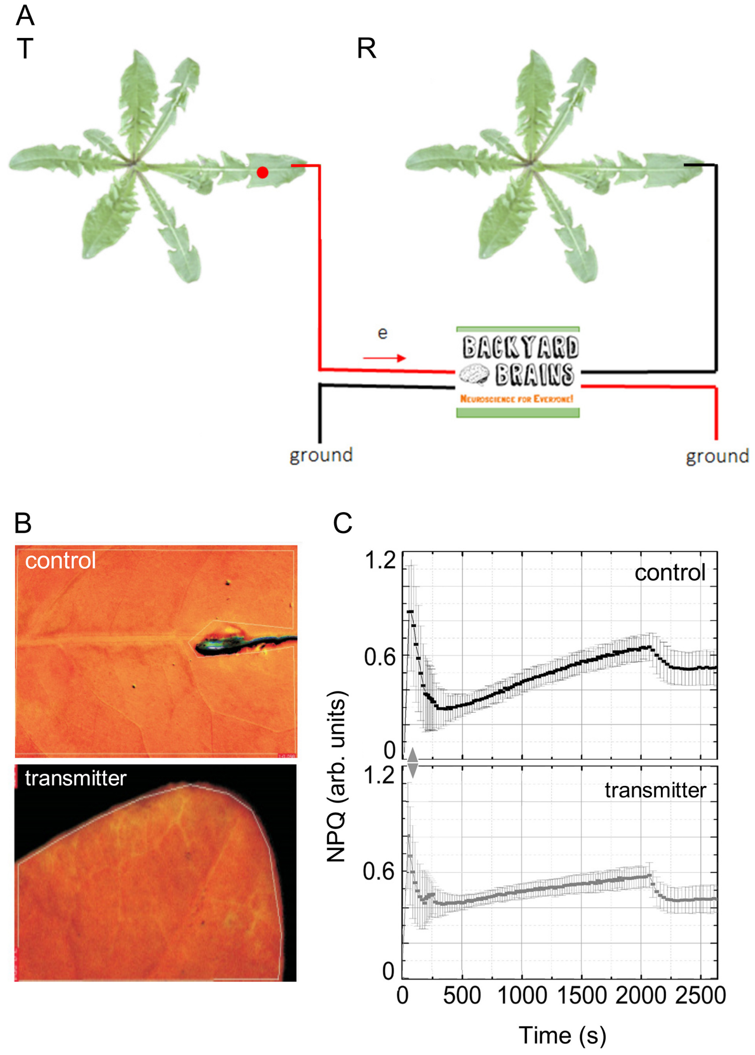

Supplemental Figure S6. Communication between two dandelion plants connected by a wire. (A) Schematic diagram of the experimental set consisting of a pair of conjugated plants. Stimulation of the transmitter plant (T, heat-injured with a heated metal wire, red dot) induced a response in the receiver plant (R, untreated). The current circuit was closed with the Backyard Brains equipment, to control the induction of electric potential and recording (https://backyardbrains.com/products/plantspikerbox). The electrodes were attached to the leaf surface with the gel (Spectra 360 electrode gel, salt free, Parker) supplied with the equipment. (B) The changes in NPQ were determined with an Imaging mini PAM (Walz) instrument, two leaves were measured at the same time. (C) Dynamics of NPQ changes over time after stimulation of T at the defined time point (arrow). Data are shown as means ± SE (n = 10).
Supports Figure 4.

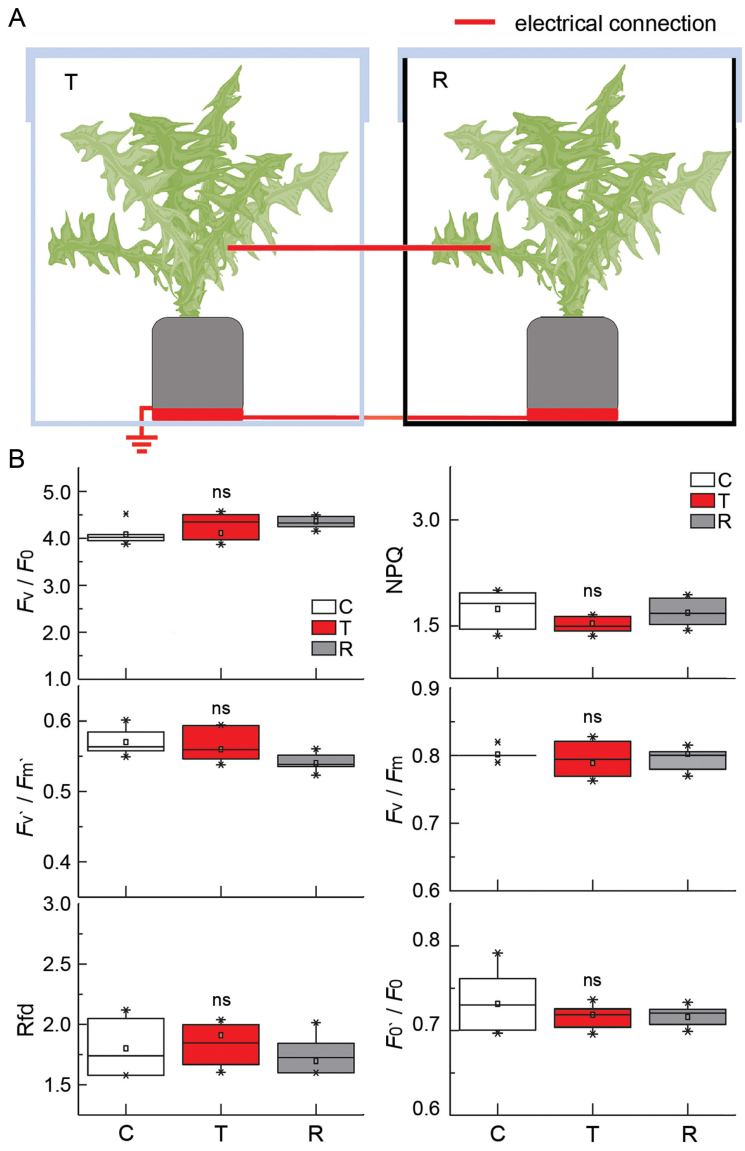

Supplemental Figure S7. Control setup for Figure 4. (A) Schematic diagram of the experimental setup used. Two different dandelion plants were placed on copper discs in separate plastic boxes, one of which was transparent and the other box had black walls. The plants and discs were coupled by copper wires threaded through the walls of the boxes. The ends of the wires coupling the plants were attached to the base of the leaves (main veins), and then the plants were grown for two days under standard laboratory conditions. Immediately prior to the experiment, the boxes were covered with transparent lids and sealed. The plants in the transparent and black boxes were untreated. (B) Chlorophyll *a* fluorescence parameters: the potential photosynthetic activity (F_v/F_0), PSII maximum efficiency ($F_{v'}/F_{m'}$), chlorophyll fluorescence decrease ratio (vitality index Rfd), nonphotochemical quenching (NPQ), the maximum quantum yield of PSII photochemistry in the dark-adapted state (F_v/F_m), fluorescence intensity ratio ($F_{0'}/F_0$) (n=10). C, control; T, transmitter; R, receiver. ns, not significant, as determined with Tukey's test.

Supports Figure 4.

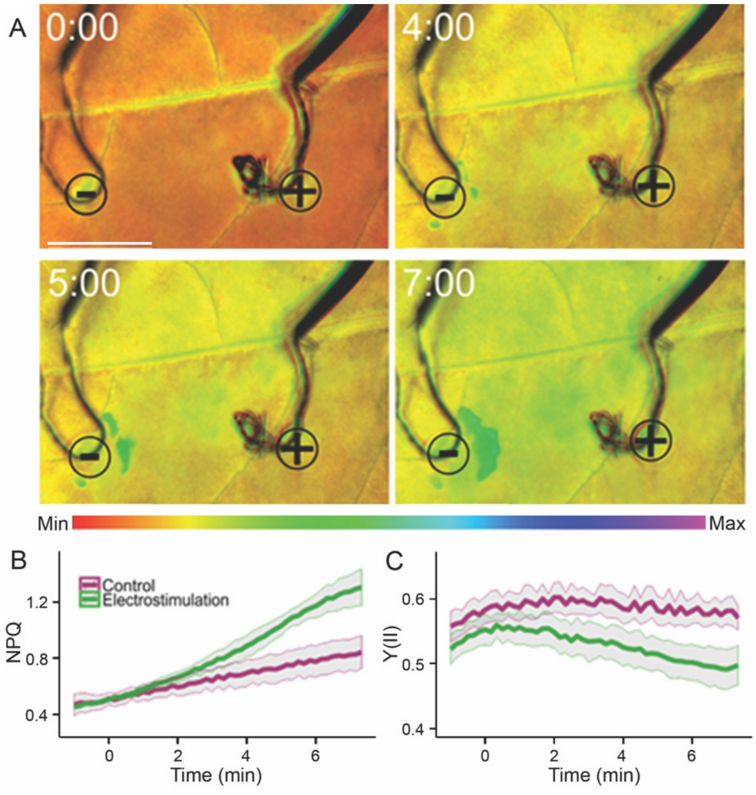

Supplemental Figure S8. Spatiotemporal changes in NPQ and yield of PSII efficiency (Y(PSII)) after electrical treatment. (A) Chl *a* fluorescence parameters of dandelion leaf were measured after treatment with the external potential difference (flat battery, 4.5 V). Experiment time is shown in min, electric stimulation was performed at 0:00. The positive terminal was the cathode (+) and the negative terminal was the anode (–). The terminal marked negative was the source of electrons. (B, C) Quantification of changes in the NPQ and Y(PSII) parameters, respectively. Data are shown as means ± SE (n=15).
Supports Figure 4.

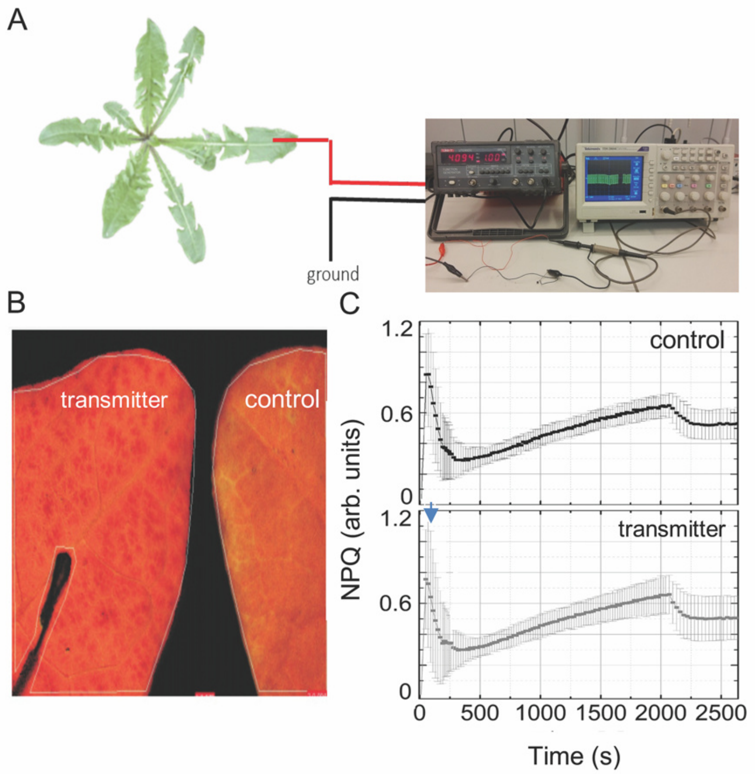

Supplemental Figure S9. Stimulation of dandelion leaves by alternating current. (A) Schematic diagram of experimental setup, consisting of a dandelion plant connected to the functional generator (UTG9005C) and oscilloscope (Tektronix TDS2004). The square wave (1V, 4Hz) was supplied through the electrodes attached to the leaf surface with gel (Spectra 360 electrode gel, salt free, Parker). (B, C) The changes in NPQ were determined with an Imaging mini PAM (Walz) instrument; only two leaves can be measured at the same time. The graph shows the dynamics of NPQ over time in the control (untreated with AC) and after stimulation of transmitter plant with AC at the defined time point (blue arrow). Data are shown as means ± SE, (n = 10).

Supports Figure 4.

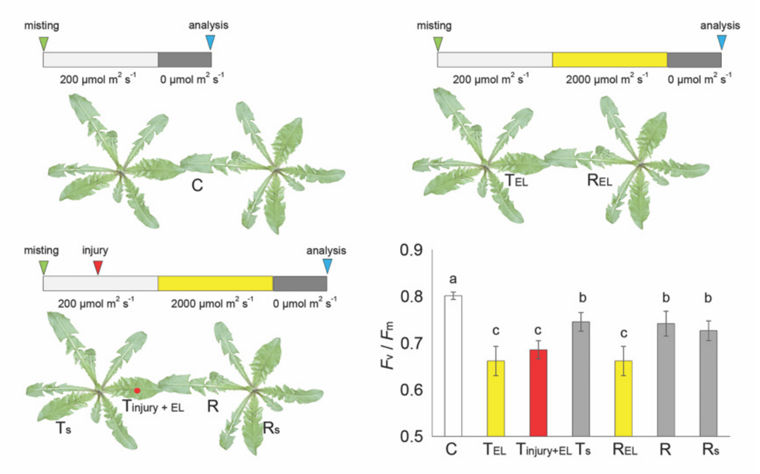

Supplemental Figure S10. Acquired acclimation responses between transmitter and receiver plants. Two different plants were placed on copper discs. Leaves of two different dandelion plants were connected by a simple touch following spraying with water to ensure conductivity. (A-C) Plants were kept in low light conditions. (B, C) Plants were treated for 1 h with excess light (2,000 µmol photons m^{-2} s^{-1}, blue 450 nm $\pm$15 nm). (C) Plants were heat-injured after 30 min of low light acclimation, and kept for another 30 min in low light conditions. (D) Photosynthetic efficiency of the different leaves. T_{EL}/R_{EL}, excess light treated plants, $T_{EL + injury}$, excess light treated and injured plants; R, the leaf on the receiver plant that touches the transmitter plant; T_s/R_s, the leaves on the transmitter/receiver plant other than those touching the receiver/ transmitter plant. Different lowercase letters indicate significant differences, as determined with Turkey's test (n=12).
Supports Figures 2, 4, 5 and Table 1.

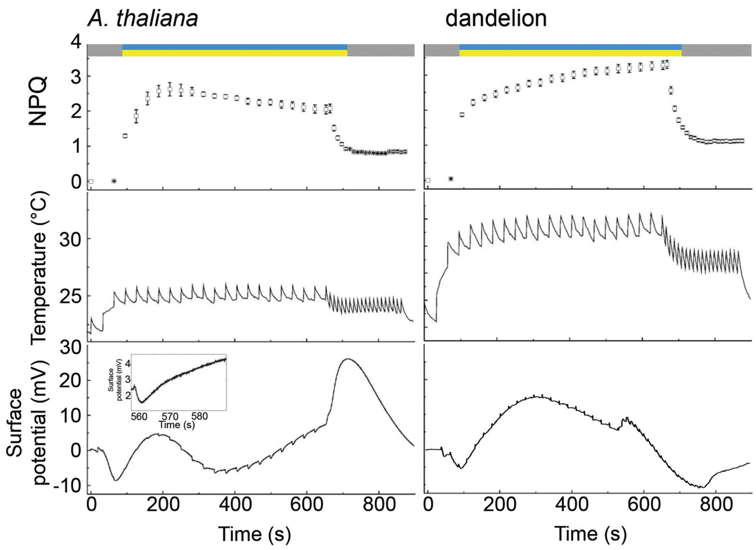

Supplemental Figure 11. Physiological changes of Arabidopsis and dandelion leaves following light treatment. Spatiotemporal change of NPQ, temperature, and electric signals detected at the surface of leaf were measured simultaneously (normalized records). Leaves were treated as follows: sudden transition from darkness (black rectangular) to blue actinic light (blue rectangular), the application of saturating pulses (yellow rectangular), darkness (black rectangular).

Supports Figures 2 and 3.

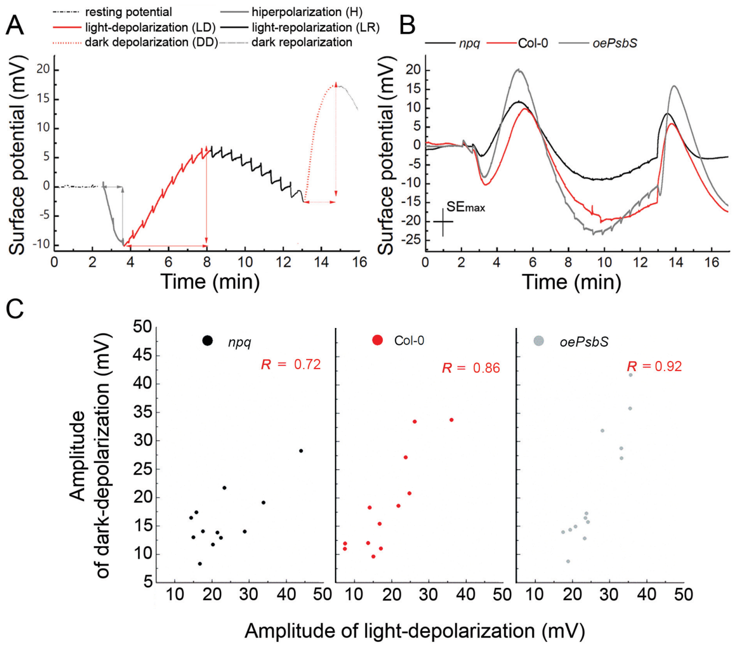

Supplemental Figure S12. ES generation in Arabidopsis Col-0, *npq*, and *oePsbS* plants. Plants were grown in low-light conditions (LL; 100 µmol photons m^{-2} s^{-1}), and then were exposed to excess light (1,500 µmol photons m^{-2} s^{-1}) applied to a local leaf. The light was switched off after 13 min. (A) The changes in the potential generated on the surface of leaf induced by the variable intensity of light: resting potential (black dashed dot thin line), hyperpolarization (thick black solid line), light depolarization (red solid line), light repolarization (thick solid black line), dark depolarization (red dashed line) and dark repolarization (gray dashed thin line); the arrows indicate the maximum amplitude of the hyperpolarization, time, and speed of this process (grey arrows), the maximum amplitude of the light depolarization, time, and speed of this process (red arrows); the maximum amplitude of the dark depolarization, time, and speed of this process (red dotted arrows). (B) Representative changes in electrical signals detected at the surface of the leaf (normalized records). Maximal SE is indicated for the x-axis (time) and the y-axis (surface potential) by the length of crossed error bars. (C) Amplitudes of dark and light depolarization from individual experiments (n=12); ρ, Pearson's correlation coefficient.
Supports Figures 2 and 3.

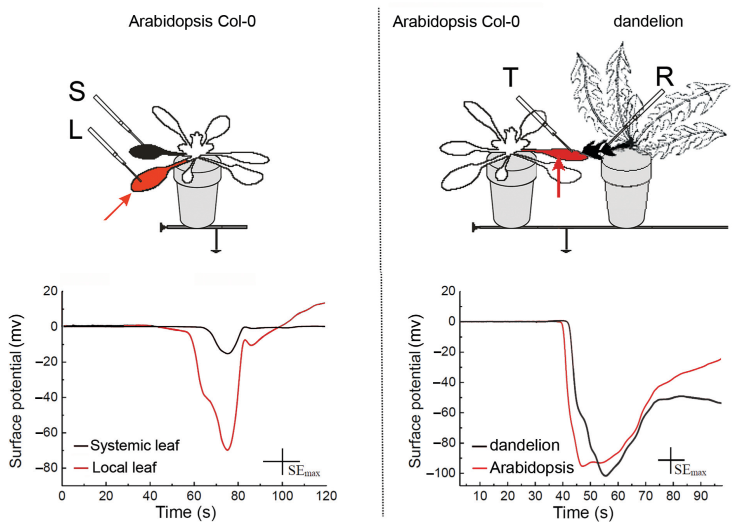

Supplemental Figure S13. ES generation in one Arabidopsis plant and ES signal transduction between one Arabidopsis plant and one dandelion plant after heat injury. Plants were grown in low-light conditions (LL; 100 µmol photons m^{-2} s^{-1}). Heat injuries were applied to a local Arabidopsis leaf (L, T). ES was transduced from the local Arabidopsis leaf (L) to a systemic leaf (S) of the same plant and from an Arabidopsis transmitter leaf (T) to a dandelion receiver leaf. Measurements were made simultaneously in leaves with two microelectrodes (normalized records). Maximal SE is indicated for the x-axis (time) and the y-axis (surface potential) by the length of crossed error bars.

Supports Figures 2 and 3.

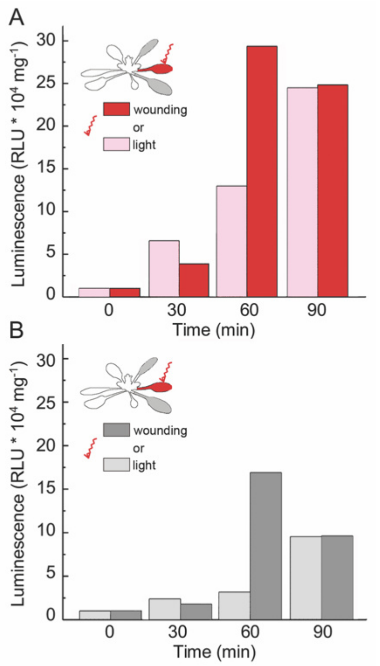

Supplemental Figure S14. SAA signal transduction between two leaves of one plant induces gene response. Arabidopsis transgenic plants harboring the *ZAT12pro:LUC* transgene expressing the luciferase (*LUC*) reporter gene under the control of the *ZAT12* promoter were grown in low-light conditions (LL; 100 µmol photons m^{-2} s^{-1}). **(A)** Induction of gene response after heat injury with a wire (wounding) or laser pointer light (2,000 µmol photons m^{-2} s^{-1}) applied to a local leaf. **(B)** Induction of gene response in an untreated systemic leaf.
Supports Figure 6.

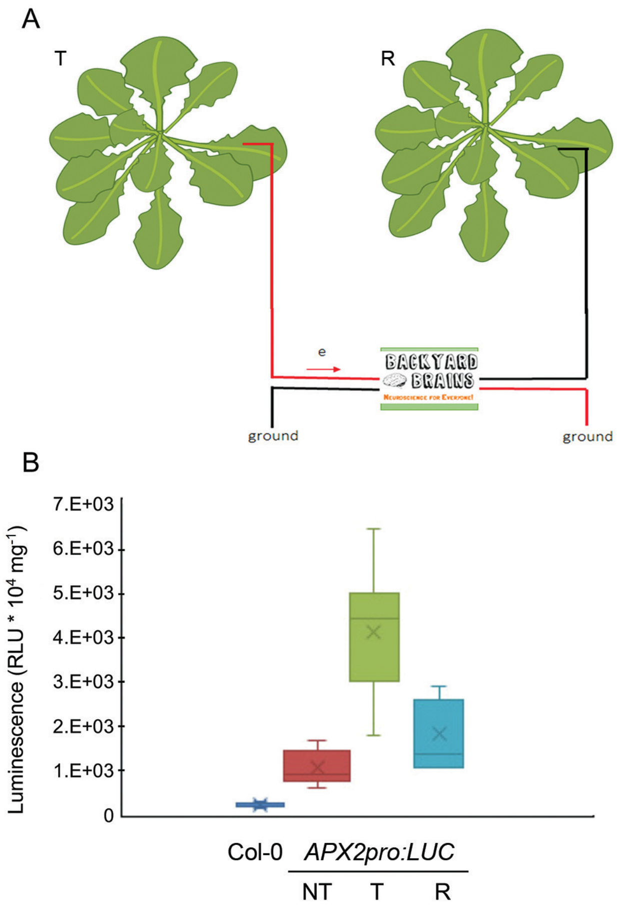

Supplemental Figure S15. SAA signal transduction between two different plants induces gene response. Arabidopsis transgenic plants carrying the *APX2pro:LUC* transgene expressing the luciferase (*LUC*) reporter gene under the control of the *APX2* promoter and Col-0 (wild type) plants were grown in low-light conditions (LL; 100 μmol photons m^{-2} s^{-1}). (A) The systems consisted of two plants connected through the Backyard Brains equipment (https://backyardbrains.com/products/plantspikerbox) and a metal wire. The ends of the wires coupling the plants were attached to the main veins of the dandelion leaf and petiole of the mimosa using the gel (Spectra 360 electrode gel, salt free, Parker) supplied with the equipment. (B) Gene expression in untreated Col-0 plant, untreated *APX2pro:LUC* plant (NT), transmitter *APX2pro:LUC* plant (T) treated with a laser pointer light (2,000 μmol photons m^{-2} s^{-1}) and in a receiver *APX2pro:LUC* plant (R, untreated). Data are shown as means ± SE (n=13).
Supports Figure 6.

Supplemental Table S1. Mean value of NPQ, FPSII and qP as a function of distance from injury (5, 10, 15, 20, and 25 mm). The results are from Figure 1.

Distance	NPQ_{max}	SE (NPQ)	$TNPQ_{max}$	SE ($TNPQ_{max}$)	FPSII	SE (FPSII)	qP	SE (qP)
5	1.079	0.174	470	27	0.392	0.024	0.601	0.034
10	0.925	0.167	474	25	0.419	0.008	0.627	0.02
15	0.937	0.152	486	16	0.4192	0.010	0.629	0.032
20	0.847	0.08	511	31	0.418	0.013	0.631	0.033
25	0.909	0.140	547	54	0.4178	0.016	0.632	0.036

T NPQ_{max}, time for reaching NPQ_{max}, in s.
SE, standard error

Supplemental Table S2. Dark relaxation of NPQ. The leaves of two different dandelion plants were connected by a drop of agarose to ensure physical contact. Plants were placed on one copper plate; however, the roots of the individual plants were separated from each other. Plants were watered well to obtain a closed-circuit system. Heat injury was applied at 330 s. Actinic light was applied over 1,630 s, and then light was switched off for the next 300 s (similar to Supplemental Figure S4). Dark relaxation of NPQ was recorded during dark treatment for the transmitter (T, n=39), receiver (R, n=35), and control plants (C, n= 31). ***, $p < 0.001$ indicate significant differences between the control and transmitter and between the control and receiver, as determined by Student's t-test.
The results are from Supplemental Figure S3, C, F.

	**Mean t (s)**	**SE (t)**
**Control**	62	2
**Transmitter**	25 ***	2
**Receiver**	47 ***	4

***, $p<0.001$

Supplemental Table S3. ES characteristics.

A. Amplitude for the transmitter and receiver in three different configurations: 1) transmitter-agarose-receiver, 2) transmitter-water-receiver, and 3) transmitter-dry-receiver (control).
The results are from Supplemental Figure S 5.

	**Transmitter**		**Receiver**	
	**Amplitude**	**SE**	**Amplitude**	**SE**
**Type of connection**				
**agarose**	–36.62	20.81	–12.03	13.78
**water**	–29.48	11.79	–12.58	6.70
**Non (dry)**	–35.53	15.46	–0.18	0.28

B. Gaussian function parameters fitted to the ES values for a transmitter and agarose-connected receiver.

**Type of connection**	**Transmitter**		**Receiver**	
**agarose**	**Value**	**SE**	**Value**	**SE**
$\mathbf{y_0}$	0	0	0	0
**xc**	74.40	6.80	78.01	7.98
**A**	–36.62	20.81	–12.03	13.78
**FWHM**	14.45	4.75	9.65	4.57
**Area**	–616.11	416.82	–108.63	108.77

Supplemental Movie S1. Turgor changes of the dandelion leaf touched three times with a heated metal wire. Total time 180 min, each injury every 30 min.
Supports Supplemental Figure S1, E-G.

Supplemental Movie S2. Signal transduction between dandelion plants under laboratory conditions. NPQ was recorded for pairs of leaves growing on two different plants that were sprayed with water. Point heat injury induced an increase in NPQ in both the transmitter (injured) and receiver (non-injured) plant. **Supports Figure 2.**

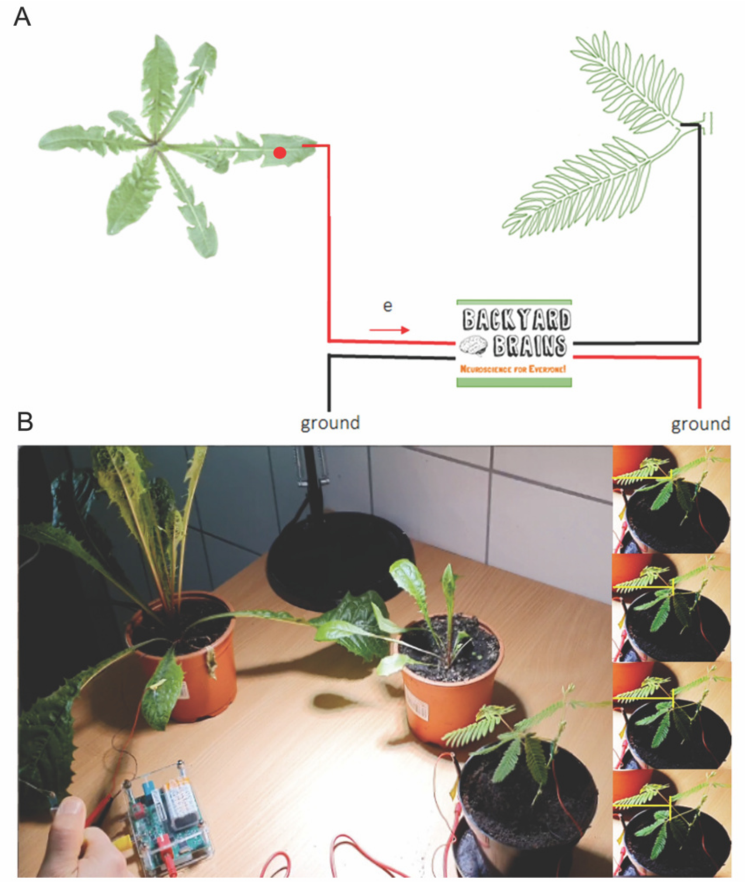

Supplemental Movie S3. Signal transduction between plants of two different species after 1-2 seconds of heat injuring treatment. (A) The system consisted of a heat-injured dandelion plant (transmitter, red dot representing the area of injury, the time period of injury treatment was 1-2 s) connected through a metal wire with an untreated mimosa plant (receiver) and the Backyard Brains equipment (https://backyardbrains.com/products/plantspikerbox). The ends of the wires coupling the plants were attached to the main veins of the dandelion leaf and the petiole of mimosa using gel (Spectra 360 electrode gel, salt free, Parker) supplied with the equipment. (B) Series of photos presenting important time points.
Supports Figure 4.

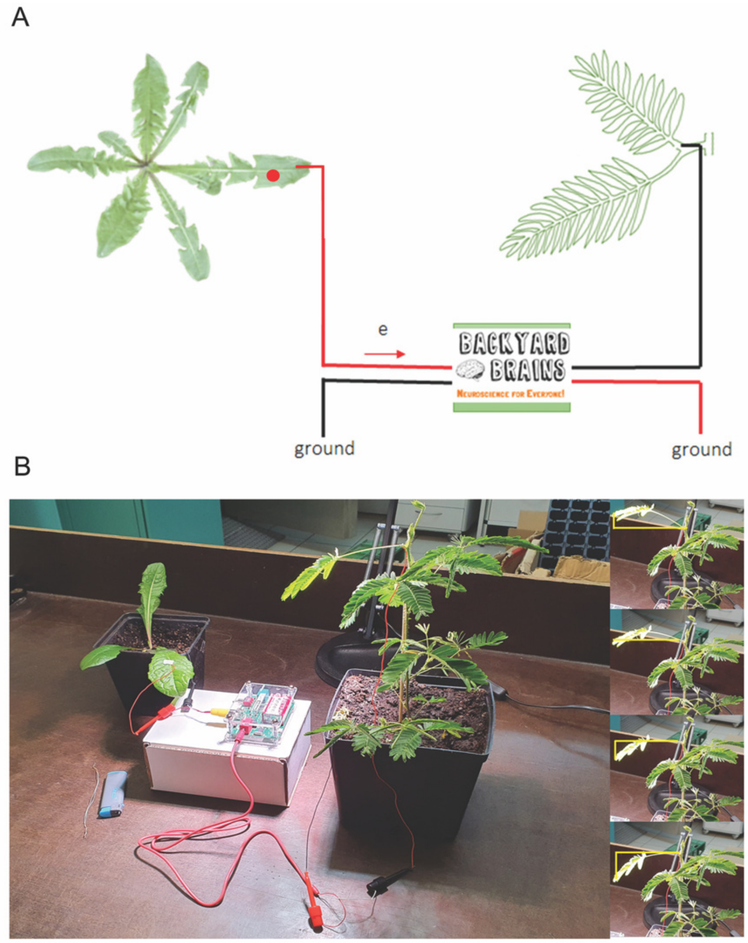

Supplemental Movie S4. Signal transduction between plants of two different species after 5 seconds of heat injuring treatment. (A) The system consisted of a heat-injured dandelion plant (transmitter, red dot representing the area of injury, the time period of injury treatment was 5 s) connected through a metal wire with an untreated mimosa plant (receiver) and the Backyard Brains equipment (https://backyardbrains.com/products/plantspikerbox). The ends of the wires coupling the plants were attached to the main veins of the dandelion leaf and the petiole of mimosa using gel (Spectra 360 electrode gel, salt free, Parker) supplied with the equipment. (B) Series of photos presenting important time points.
Supports Figure 4.

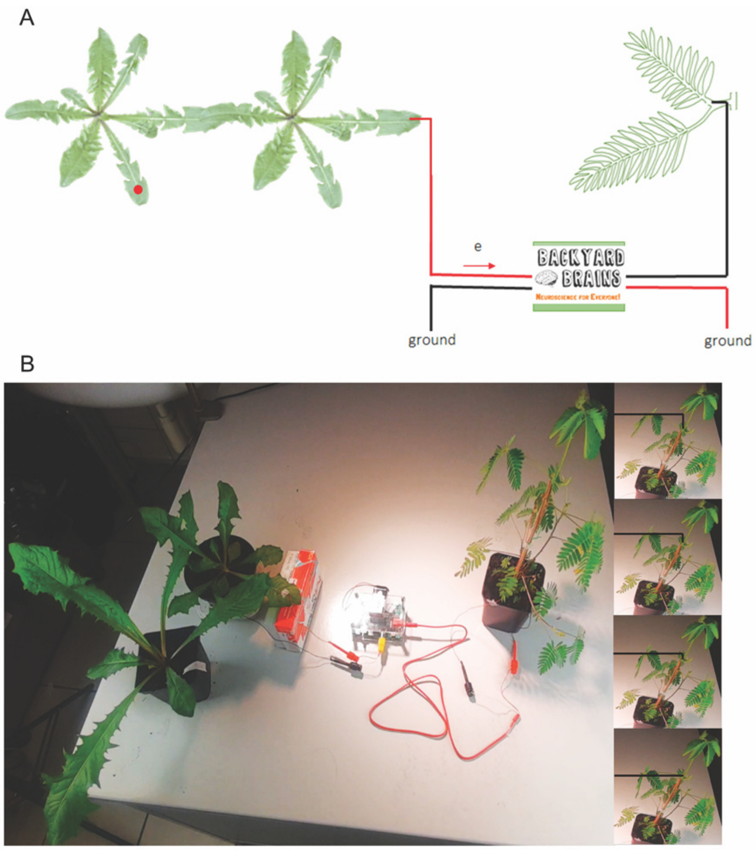

Supplemental Movie S5. NAA signal transduction between three plants of two different species after 1 second of heat injury treatment. (A) The system consisted of a heat-injured dandelion plant (primary transmitter, the red dot represents the area of injury, the time period of injury treatment was 1-2 s), touching another untreated dandelion plant (secondary transmitter), and then the secondary transmitter was connected to an untreated mimosa plant (receiver) through a metal wire and the Backyard Brains equipment (https://backyardbrains.com/products/plantspikerbox). The ends of the wires coupling the plants were attached to the main veins of the dandelion leaf and the petiole of mimosa using gel (Spectra 360 electrode gel, salt free, Parker) supplied with the equipment. (B) Series of photos presenting important time points.
Supports Figure 4.

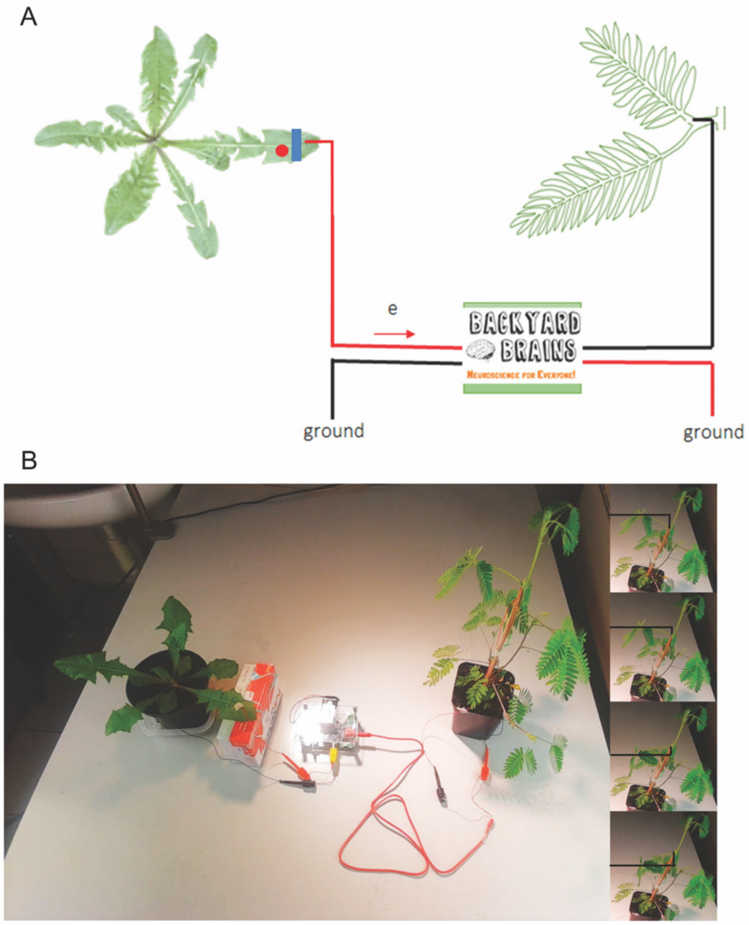

Supplemental Movie S6. Signal transduction between plants of two different species treated with $LaCl_3$. The systems consisted of a dandelion plant treated with lanthanum chloride ($LaCl_3$, calcium channel blocker) and then touched with a heated metal wire (transmitter, red dot represents the area of touch, the time period of touch was 1-2 s, the blue rectangular represents the area of $LaCl_3$ treatment). The transmitter was connected to an untreated mimosa plant (receiver) and Backyard Brains equipment (https://backyardbrains.com/products/plantspikerbox). The ends of the wires coupling the plants were attached to the main veins of the dandelion leaf and petiole of the mimosa using gel (Spectra 360 electrode gel, salt free, Parker) supplied with the equipment.

Supports Figure 4.

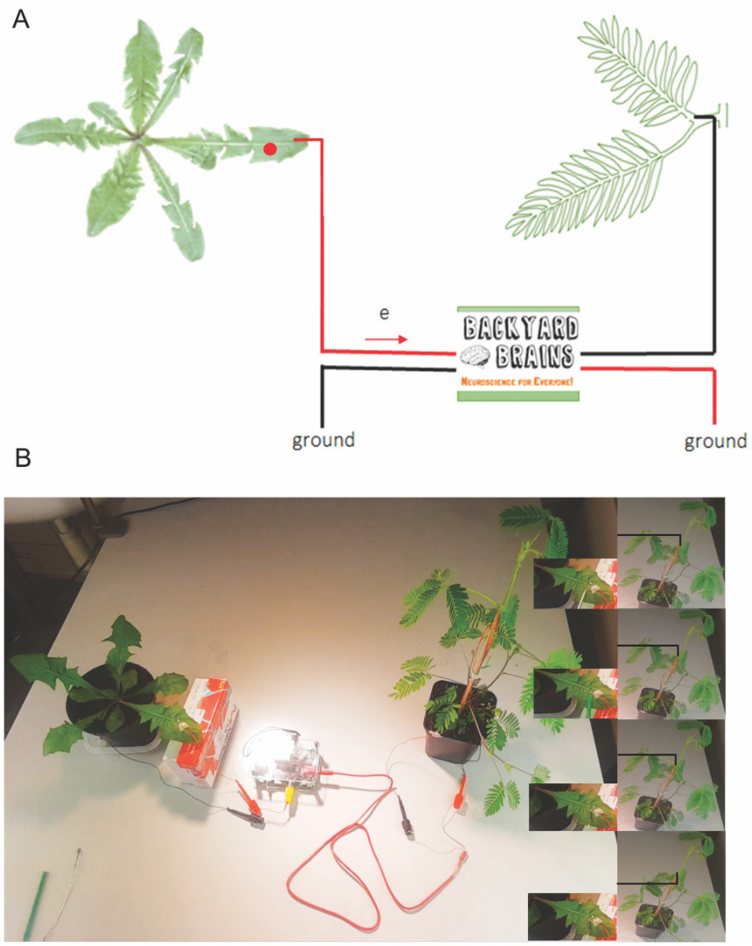

Supplemental Movie S7. Lack of signal transduction between plants of two different species toughed with unheated plastic stick, wood stick, and metal wire. The systems consisted of a dandelion plant touched with an unheated plastic stick, then an unheated wood stick, and finally an unheated metal wire (transmitter, red dot represents an area of the touch). The time period of touch was 1-2 s. The transmitter was connected to an untreated mimosa plant (receiver) and Backyard Brains equipment (https://backyardbrains.com/products/plantspikerbox). The ends of the wires coupling the plants were attached to the main veins of the dandelion leaf and petiole of the mimosa using gel (Spectra 360 electrode gel, salt free, Parker) supplied with the equipment.
Supports Figure 4.

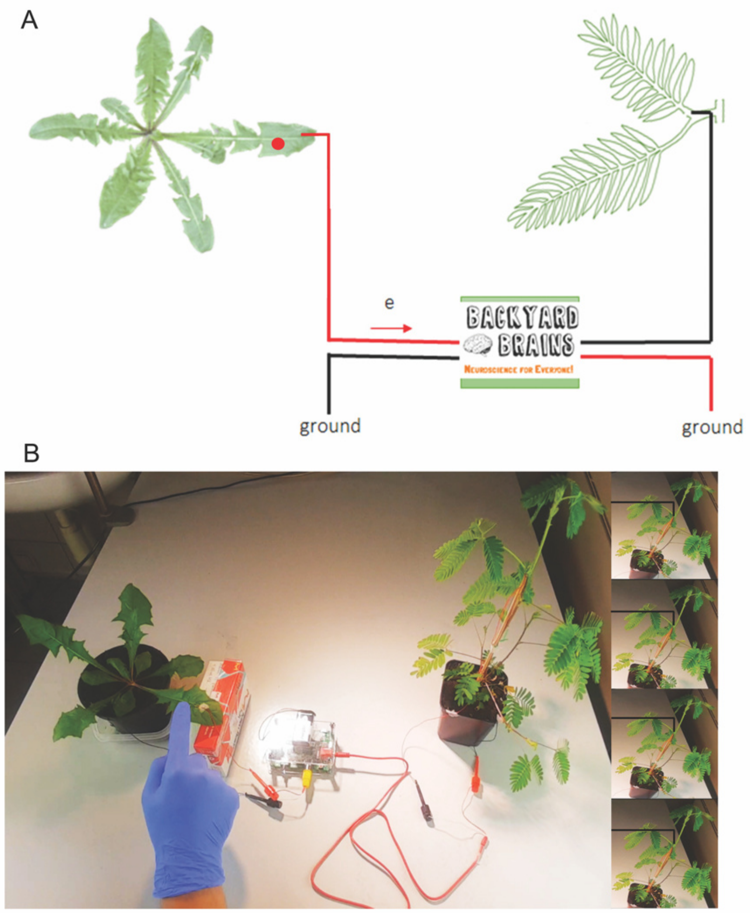

Supplemental Movie S8. Lack of signal transduction between plants of two different species touched with a finger in a rubber glove. The systems consisted of a dandelion plant touched with a finger in a rubber glove (transmitter, red dot representing the area of touch). The time period of touch was 1-2 s. The transmitter was connected to an untreated mimosa plant (receiver) and Backyard Brains equipment (https://backyardbrains.com/products/plantspikerbox). The ends of the wires coupling the plants were attached to the main veins of the dandelion leaf and petiole of the mimosa using gel (Spectra 360 electrode gel, salt free, Parker) supplied with the equipment.
Supports Figure 4.

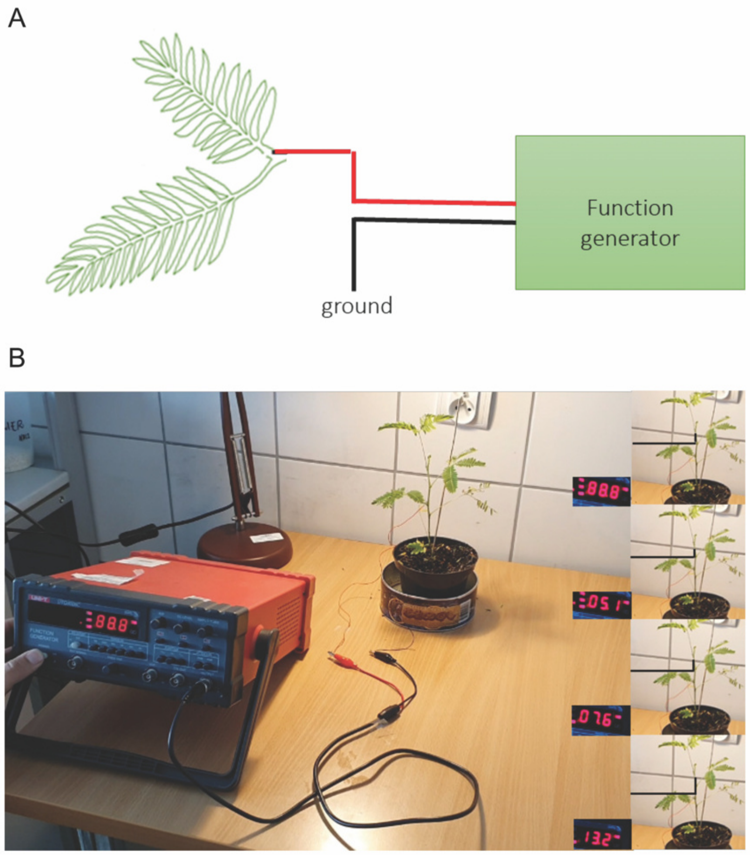

S

upplemental Movie S9. Electrical stimulation with an alternating current (0-133 V AC). (A) Schematic diagram of the system consisting of a mimosa plant treated with 0.00-133 V AC (4 Hz), which was supplied as square waves from a function generator (UTG9005C). The electrode was attached to the leaf petiole with gel (Spectra 360 electrode gel, salt free, Parker). (B) Series of photos presenting important time points. Note: In the first part of the movie, the thigmonastic responses were not induced when the mimosa plant was treated with 51 V AC. In the second part of the movie, the mimosa plant was treated with 51-133 V AC, and the 70 V AC was a threshold above which thigmonastic responses were induced.
Supports Figure 4.

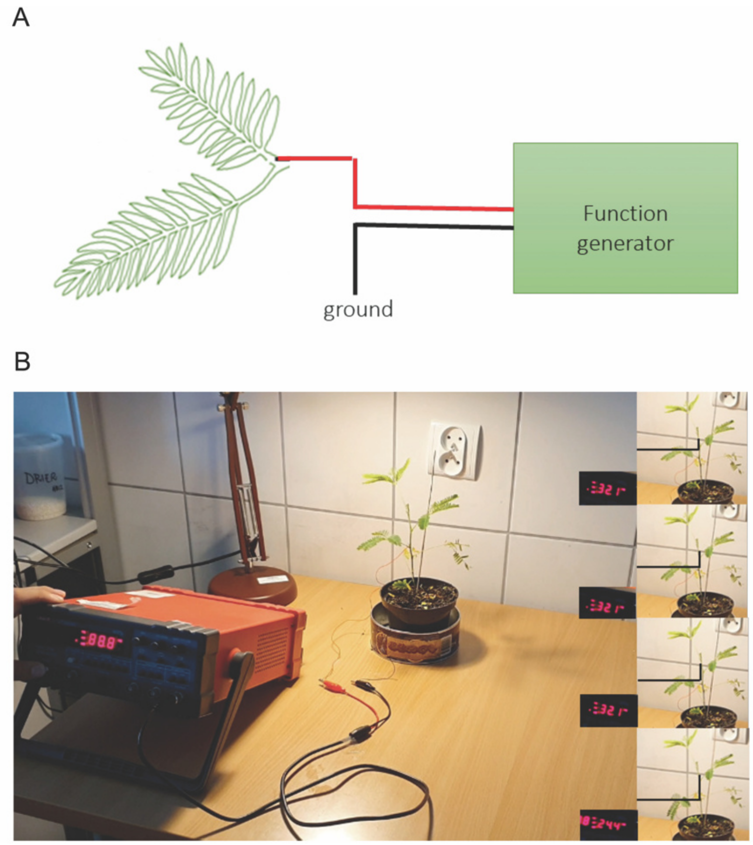

Supplemental Movie S10. Electrical stimulation with an alternating current (~240 V AC). (A) Schematic diagram of the system consisting of a mimosa plant treated with ~240 V AC (4 Hz), which was supplied as square waves from a function generator (UTG9005C). The electrode was attached to the leaf petiole with gel (Spectra 360 electrode gel, salt free, Parker). (B) Series of photos presenting important time points.
Supports Figure 4.
